# Evaluation of Possible Antioxidant, Anti-Hyperglycaemic, Anti-Alzheimer and Anti-Inflammatory Effects of *Teucrium polium* Aerial Parts (Lamiaceae)

**DOI:** 10.3390/life12101579

**Published:** 2022-10-11

**Authors:** Naima Benchikha, Mohammed Messaoudi, Imane Larkem, Hamza Ouakouak, Abdelkrim Rebiai, Siham Boubekeur, Mohamed Amine Ferhat, Adel Benarfa, Samir Begaa, Mokhtar Benmohamed, Diena M. Almasri, Rawan H. Hareeri, Fadia S. Youssef

**Affiliations:** 1 Laboratory of Applied Chemistry and Environment (LCAE), Chemistry Department, University of Hamma Lakhdar El-Oued, B.P.789, El-Oued 39000, Algeria; 2 Nuclear Research Centre of Birine, P.O. Box 180, Ain Oussera, Djelfa 17200, Algeria; 3 Laboratory of Diversity of Ecosystems and Dynamics of Agricultural Production Systems in Arid Zones, Department of Agronomy, Faculty of Nature and Life Science, Biskra University, Biskra 07000, Algeria; 4 Research and Development Centre RDC-SAIDAL, 35Benyoucef Khattab Avenue, Mohammadia, El-Harrah, Algiers 16000, Algeria; 5 Ecole Normale Supérieure (ENS), P.O. Box 92, Vieux-Kouba, Alger 16308, Algeria; 6 Centre de Recherche Scientifique Et Technique en Analyses Physico-Chimiques (CRAPC)-PTAPC, P.O. Box 0354, Laghouat 03000, Algeria; 7 Laboratory of Fundamental Sciences, University Amar Télidji of Laghouat, P.O. Box 37G, Road of Ghardaïa, Laghouat 03000, Algeria; 8 Department of Pharmacy Practice, Faculty of Pharmacy, King Abdulaziz University, Jeddah 21589, Saudi Arabia; 9 Department of Pharmacology and Toxicology, Faculty of Pharmacy, King Abdulaziz University, Jeddah 21589, Saudi Arabia; 10 Department of Pharmacognosy, Faculty of Pharmacy, Ain-Shams University, Abbasia, Cairo 11566, Egypt

**Keywords:** *Teucrium polium* L., antioxidant, anti-hyperglycemic, anti-Alzheimer, anti-inflammatory, molecular docking, drug discovery, health care

## Abstract

*Teucrium polium* L. is commonly used in folk medicine to treat hypertension and diabetes and to heal wounds. The present work aimed to evaluate the different biological activities of *T. polium* hydroalcoholic extract, its total phenol and flavonoid content, and its mineral elements. Results showed that *T. polium* extract showed significant antioxidant potential in 2-diphenyl-1-picrylhydrazyl (DPPH) assay with IC_50_ equal to 8.68 μg/mL but with moderate activity in galvinoxyl assay with IC_50_ of 21.82 μg/mL and mild activity in the *β*-carotene assay. It also showed a pronounced anti-hyperglycemic activity using α-amylase inhibitory assay (IC_50_ = 111.68 µg/mL) and exceeds that of acarbose. *T. polium* showed excellent activity against acetylcholinesterase (AChE) and butyrylcholinesterase (BChE) with IC_50_ values of 28.69 and 4.93 μg/mL, respectively, postulating its promising anti-Alzheimer potential. The plant extract exhibited a strong anti-inflammatory effect with Bovine Serum Albumin (BSA) denaturation inhibitory potential estimated by 97.53% at 2 mg/mL, which was further confirmed by the in vivo carrageen-induced edema model. The extract revealed its richness in flavonoids and phenols, evidenced by its polyphenols content (36.35 ± 0.294 μg GAE/mg) and flavonoids (24.30 ± 0.44 μg QE/mg). It is rich in minerals necessary for human health, such as calcium, potassium, iron, sodium, magnesium, manganese and zinc. Molecular docking performed for previously identified compounds on human *α*-amylase, 5-lipoxygenase (5-LOX) and acetylcholine esterase confirmed the results. Thus, it can be concluded that *T. polium* can be a good candidate for alleviating many health-debilitating problems and can be highly beneficial in the pharmaceutical industry and medical research.

## 1. Introduction

Oxidative stress has been recognized as the main cause of many life-threatening diseases, comprising atherosclerosis, cardiovascular diseases, diabetes, obesity, cancer, and many inflammatory conditions [1,2]. The unregulated inflammatory responses further worsen the previously mentioned disorders that could be managed via the consumption of steroidal or non-steroid anti-inflammatory and immunosuppressant drugs. However, they trigger a plethora of side effects [3,4]. In addition, diabetes mellitus and Alzheimer’s disease (AD) are among the most debilitating disorders that greatly influence patients’ capabilities causing a significant reduction in their activities and affecting their well-being [5].

Naturally occurring antioxidants of plant origin are highly popular for combatting oxidative stress and counteracting inflammation and its associated disorders, owing to their richness with bioactive secondary metabolites [6,7,8,9]. Furthermore, they have the potential to effectively inhibit oxidative stress that is associated with diseases such as diabetes and neurodegenerative disorders [10]. As a result, they are highly welcomed by a large category of patients all over the globe and, thus, can be used as an alternative to synthetic drugs due to their lower adverse effects and price compared to synthetic ones [11,12]. The pronounced anti-inflammatory potential of medicinal plants is mainly attributed to their richness in natural antioxidants, such as flavonoids, polyphenols, tocopherols, carotenoids, and ascorbic acid [1,13].

Genus *Teucrium* belonging to the family Lamiaceae includes about 300 species greatly spread throughout North Africa, Europe and Asian temperate regions [14], where its different species showed many activities [15,16]. *Teucrium polium* L. is widely used in traditional medicine to treat hypertension and diabetes or as a wound-healing agent [13]. *T. polium* is a deciduous shrub native to the Western Mediterranean region [17]. It showed many biological activities, such as anti-inflammatory, antiviral, antifungal, antibacterial, cytotoxic, antioxidant, hypoglycemic, hypolipidemic, hepatoprotective, analgesic, antiulcer effects, in addition to anticonvulsant potential. These activities are highly attributed to plants’ bioactive secondary constituents, such as phenylethanoid glycosides, flavonoids, diterpenes, iridoids and essential oil [13,14,17].

In this study, we aimed to evaluate the antioxidant, anti-hyperglycaemic, anti-Alzheimer and anti-inflammatory effects of *Teucrium polium* hydroalcoholic extract from the aerial parts. The antioxidant was assessed using 2-diphenyl-1-picrylhydrazyl (DPPH), β-carotene and Galvinoxyl radical (GOR) assays, whereas the anti-hyperglycemic was determined in vitro by the α-amylase inhibition method. The anti-inflammatory effect was determined in vitro by inhibiting denaturation of BSA (Bovine Serum Albumin) and in vivo by inhibiting mouse paw edema induced by carrageenan. However, the anti-Alzheimer activity of the extract of *T. polium* aerial parts was assessed in vitro via the determination of the anti-cholinesterase activity carried out by the acetylcholinesterase inhibitor method (AChE) and butyrylcholinesterase (BChE). Total phenol and flavonoid content, as well as mineral contents, were evaluated for the first time. Moreover, the correlation between the studied activities and its major previously identified metabolites was determined in silico using molecular docking within the active sites of human *α*-amylase (HA), acetylcholine esterase and 5-lipoxygenase using Discovery Studio 4.5 (Accelrys Inc., San Diego, CA, USA) with C-docker protocol to further consolidate the obtained results. This work is part of an overall program in our laboratory to use nuclear analytical techniques for studying natural food samples relevant to human health and nutrition.

## 2. Materials and Methods

### 2.1. Reagents and Materials

Folin-Ciocalteu (FCR), sodium carbonate (Na_2_CO_3_),2,2-diphenyl-1-picrylhydrazyl (DPPH), 2,2′-azino-bis(3-ethylbenzothiazoline-6-sulfonic acid) ABTS, α-Tocopherol (Vitamin E), 2,6-ditert- butyl-4-methylphenol (BHT), gallic acid, quercetin, and AlCl_3_ were purchased from Sigma (Sigma-Aldrich, Germany). All the organic solvents and other chemicals used in the present study were of analytical grade and were obtained from Sigma-Aldrich (Sigma Aldrich, Germany).

### 2.2. Plant Material

The aerial parts of *Teucrium polium* L. (Lamiaceae family) were collected on June 2018 from the El-Guetfa region, Msila, located in the semi-arid region of Algeria (35°44′26 N and 3°23′05 E). The plant material was identified taxonomically based on the identification methods described by Quezel and Santa [18] and by one of the authors, H.O., and Professor A. Hassani (ENS-kouba). A voucher sample was stored in the Chemistry Department, University of Hamma Lakhdar El-Oued, Algeria, under the code (RO-050). The aerial parts were cleaned and dried. Dry aerial parts were pounded with an electric blender and kept wrapped in paper until use.

### 2.3. Preparation of the Plant Hydroalcoholic Extract

The aerial parts of *T. polium* L. samples were washed several times with deionized water and dried for two weeks at room temperature. The dried samples were ground to a fine powder (particle size fraction of <200 µm) using an agate mortar and pestle. After that, 20 g of plant powder was soaked in a solvent fraction of ethanol/water (80/20) (%) for 24 h, then filtered and dried in a rotary vapor under reduced pressure at 45 °C. The extraction yield was calculated and found to be 20.45%

### 2.4. Determination of the Biological Activity of T. polium Hydroalcoholic Extract

#### 2.4.1. Evaluation of Possible Antioxidant Activity In Vitro

The antioxidant activity of *T. polium* L. hydroalcoholic extract was achieved using several analytical tests distinguished by their mechanism of reaction 2-diphenyl-1-picrylhydrazyl (DPPH), β-carotene and galvinoxyl radical (GOR). The reaction was initiated by mixing 0.5 mL of hydroalcoholic extract with 1 mL of DPPH (0.2 mM), β-carotene (0.5 mM) and GOR (0.1 mM) using the protocols described in references [19,20]. The mixtures were left in the dark at room temperature for approximately 30 min; after that, the absorbance was measured at λ_max_: 596, 490 and 428 nm for the mentioned tests, respectively, against a blank using a UV-vis spectrophotometer apparatus (Shimadzu UV-2450, Japan). The inhibition percentage was calculated as follows: I% = ((A_0_ − A_1_)/A_0_) × 100, where A_0_ is the absorbance of DPPH, β-carotene and GOR solutions without extracts, while A_1_ were the absorbance of the sample. The activity results for all tests were expressed as IC_50_ (half maximal inhibitory concentration) values, which have been calculated (using Microsoft Excel 2016) from an antiradical linear graph formed by the X axis, which represented extract concentration, and the Y axis, which represented their relative inhibition percentage. In addition, the antioxidant activity results were compared with three standards, BHA, BHT and vitamin, as represented in Table 1.

#### 2.4.2. Evaluation of Possible Anti-Hyperglycemic Activity In Vitro

The anti-hyperglycemic activity of the *T. polium* aerial parts hydroalcoholic extract was tested using the α-amylase inhibition method according to the Zengin method with some modifications [21]. On a 96-well microplate reader (Perkin Elmer EnSpire, Singapore), a volume of 25 µL of the hydroalcoholic extract at different concentrations (62.5, 125, 250, 500 and 4000 with an increment of µg/mL) was mixed with 50 µL of 1U α-amylase solution and then incubated for 10 min at 37 °C. Then 50 μL of starch (0.1%) was added. The mixture was incubated again for 10 min at 37 °C. After incubation, 25 μL of hydrochloric acid (1 M) (prepared by diluted 4.17 mL of pure HCl with 45.83 mL distilled water) and 100 μL of iodine-potassium iodide solution was added. The absorbance was read at 630 nm. Acarbose was used as a standard. The following formula was used to calculate α-amylase inhibition percentage: I% = 1 − [(A_c_ − A_e_) − (A_s_ − A_b_)/(A_c_ − A_e_)]

Ac = Absorbance [Starch + IKI + HCL + Extract solvent volume + Enzyme buffer volume]

Ae = Absorbance [Enzyme + Starch + IKI + HCl + Vol. extract solvent]

As = Absorbance [Enzyme + Extract + Starch + IKI + HCl]

Ab = Absorbance [Extract + IKI + 125 µL of buffer]

#### 2.4.3. Evaluation of Possible Anti-Alzheimer Activity In Vitro

The anti-Alzheimer activity of the hydroalcoholic extract of *T. polium* aerial parts was assessed in vitro via the determination of the anti-cholinesterase activity carried out by the method of acetylcholinesterase inhibitor (AChE) and butyrylcholinesterase (BChE). This was measured using the spectrophotometric method previously reported by Ellman with slight modifications [22]. AChE from electric eel and BChE from horse serum were used, while acetylthiocholine iodide (ACI) and butyrylthiocholine chloride (BuCi) were used as reaction substrates. DTNB solution (5,5′-Dithiobis (2-nitro-benzoic acid)) was used to measure cholinesterase activity. Briefly, 150 µL of 100 mM sodium phosphate buffer (pH = 8), 10 µL of the sample solution (4 mg/mL) dissolved in methanol at different concentrations, and 20 µL of AchE or BchE solution prepared in a buffer (pH = 8) were mixed and incubated for 15 min at 25 °C. Then 10 µL of D-TNB (0.5 mM) were added. The reaction was then initiated by adding 10 μL of acetylthiocholine iodide (0.71 mM) or 10 μL of butyrylthiocholine chloride (0.2 mM). The hydrolysis of these substrates was monitored spectrophotometrically at a wavelength of 412 nm using a 96-well microplate reader (Perkin Elmer EnSpire, Singapore) by forming a yellow 5-thio-2-nitrobenzoate anion resulting from the reaction of DTNB with thiocholine, released by the enzymatic hydrolysis of acetylthiocholine iodide or butyrylthiocholine chloride, respectively. Galanthamine was used as a reference compound, and the results were given as percent inhibition against concentrations of 25, 50, 100 and 200 µg/mL; the IC_50_ was also given. The percent inhibition of BChE and AChE was determined using the following formula: I% = [(E − S)/E] ∗ 100, where E: the activity of the enzyme without extract, and S: the activity of the enzyme with the extract.

#### 2.4.4. Evaluation of Possible Anti-Inflammatory Activity In Vitro

In vitro anti-inflammatory activity was determined for the hydroalcoholic extract of *T. polium* aerial parts using the inhibiting denaturation of BSA (Bovine Serum Albumin) following the method previously reported by Kandikattu et al. [23], with slight modifications. It relied upon the inhibition of the denaturation of BSA caused by heat (72 °C). Briefly, 1 mL of each concentration of extract or standard (Diclofenac sodium) was added to 1 mL of 0.2% BSA solution prepared in tris-HCl (pH = 6.6). The solutions were incubated at 37 °C for 15 min in an oven, then in a water bath at 72 °C for 5 min. After cooling, the turbidity was measured at 660 nm in a UV-visible spectrophotometer. For each concentration of extract, a blank is prepared in 1 mL of hydroalcoholic extract and 1 mL of tris-HCl (the purpose of this blank is to subtract the absorbance of the extract from the results obtained). The protective effect of samples against the denaturation of BSA was presented as inhibition percentages calculated using the following formula: I% = [(Ac − As)/As] ∗ 100, where I%: the inhibition percentage, A_C_: absorbance of the control and A_S_: absorbance of the tested sample.

#### 2.4.5. Evaluation of the Anti-Inflammatory Activity In Vivo

##### Experimental Animals

The anti-inflammatory activity was performed in vivo using albino mice of Swiss strain from the Pasteur Institute (Algiers). Male mice were used with weights ranging from 20 to 22 g. The laboratory animals were housed in plastic cages at room temperature (25 °C) and exposed to light for 12 h per day. During the acclimatization period (a week before being used in the various experiments), the mice had free access to water and food (croquettes from the Animal Feed Production Company, Bouzareah, Alger).

##### Experimental Protocol

The anti-inflammatory activity was assessed by inhibiting mouse paw edema induced by carrageenan following the protocol previously described by Colot. [24]. The principle of this study consists of injecting carrageenin under the plantar fascia of the left paw of the mouse to cause an inflammatory reaction, which can be reduced by an anti-inflammatory product. This study allows the comparison of plantar edema after administration of equal doses of the anti-inflammatory product to be tested (the hydroalcoholic extract of the plant at 10%) and the corresponding reference product (Diclofenac sodium at 10 mg/kg). The experiment was carried out as follows:

At time T_0_, the mice were divided randomly into 3 batches; each batch contained 5 mice that were made up randomly. In the control group: each mouse received 0.5 mL of physiological water. Each mouse received a reference anti-inflammatory drug, Diclofenac, in the standard group at 10 mg/kg. The treated group received the test solution, where each mouse received 0.5 mL of the plant extract at 10%, where 5 g were suspended in 50 mL H_2_O to form a 10% tested solution. Before starting the experiments, the mice fasted for 16 h, weighed and then were administered intragastrically (gavage) for the three batches of these suspensions, namely, control solution, reference and extract of the plant. Then half an hour after dosing, mice from the three batches received 0.025 mL of 1% carrageenan under the plantar fascia of the left hind paw of the mouse. Then four hours after this operation, the animals were sacrificed by cervical dislocation, the hind legs were cut at the height of the joint and then weighed on an analytical balance. The anti-inflammatory activity was calculated as a percentage reduction in edema in the treated mice compared to the control according to the following formula:

I% of edema = ((PG − PD) _control mouse_ − (PG − PD) _treated mouse_)/((PG − PD)_control mouse_)

where PD = right leg weight, and PG = left leg weight

### 2.5. Determination of the Total Content of Phenol and Flavonoids

Determination of the total content of phenol and flavonoids was obtained by a spectrophotometric method employing the Singleton–Rossi method with the Folin–Ciocalteu reagent at wavelength λ = 765 nm, using a gallic acid graph as reference for the total phenol content, whereas quercetin was used as a reference for the total flavonoid content and at the wavelength λ = 420 nm.

#### 2.5.1. Estimation of Total Phenol Content (TPC)

The total phenol content was determined using the Folin–Ciocalteu reagent [25], according to a microplate assay method described by Müller [26]. The FCR reagent, consisting of a mixture of phosphotungstic acid (H3PW_12_O_40_) and phosphomolybdic acid (H3PMo12O40), was reduced, during the oxidation of phenols, to a mixture of tungsten oxides (W_8_O_23_) and molybdenum (Mo_8_O_23_). The blue coloration is proportional to the total phenol content and has a maximum absorption of around 750–765 nm. A volume of 20 μL of the samples was added to 100 μL of Folin–Ciocalteu reagent diluted to (1:10) and 75 μL of sodium carbonate (Na_2_CO_3_) (7.5%). After 2 h of incubation at room temperature and in the dark, the absorbance of different intensities of the resulting blue color was measured at 765 nm. A blank was prepared in the same way, replacing the extract with the solvent used (ethanol/water). Gallic acid with concentrations ranging from 0 to 200 µg/mL was used to construct the calibration curve as a standard. The results were expressed in µg of gallic acid equivalent (GAE) by milligram of extract (µg EAG/mg).

#### 2.5.2. Estimation of Total Flavonoid Content (TFC)

The analysis of flavonoids in the hydroalcoholic extracts is based on the formation of a complex between Al^+3^ and the flavonoids. Topçu method [27] was used with some modifications in a 96-well microplate to determine the total flavonoid content. In total, 50 µL of the extract was mixed with 130 µL of methanol, 10 µL of potassium acetate and 10 µL of aluminum nitrate. After 40 min of incubation in the dark and at room temperature, the absorbance was read at 415 nm. The concentration of flavonoids in the extracts was calculated from a calibration curve established with quercetin at different concentrations ranging from 0 to 40 μg/mL. The results were expressed as equivalent micrograms of quercetin per milligram of extract (μg EQ/mg of extract).

### 2.6. Determination of Mineral Elements

Instrumental Neutron Activation Analysis (INAA) and inductively coupled plasma-optical emission spectrometry (ICP-OES) at Es-Salam Research reactor, Algeria, were used to determine the concentrations of the elements.

#### 2.6.1. Determination of Mineral Elements by the INAA Technique

In the INAA technique, three samples of each collected charge weighing about 200 mg were stored in pre-cleaned polyethylene capped bottles. In addition, certified reference materials, such as NIST-SRM 1573a (tomato leaves) from the National Institute of Standard and Technology (NIST) and GBW 07605 (National Research Center for CRM. Langfang, China), were used. All the samples and standards were packed and irradiated together for 6 h at a thermal neutron flux of 4.5 × 10^13^ cm^−2^s^−1^. After appropriate cooling, the irradiated samples and the standard were measured at different cooling times using a coaxial HPGe detector with the following characteristics: relative efficiency: 35%, FWHM 1.8 keV for the 1332.5 keV γ-peak of ^60^Co. In the end, the concentrations of elements (major and trace elements) were determined using the equation of INAA given by the following [28].

$$\rho \left(µg/g\right)=\frac{\;{\left[\left(N_{p}/t_{c}\right)/DC\right]}_{a}}{{\left[\left(N_{p}/t_{c}\right)/DC\right]}_{s}}\frac{{\left(\rho W\right)}_{s}\;}{W_{a}}\;$$

The subscripts *a* and *s* refer to the sample and the standard, respectively, *N_p_* is the net photo-peak counts, *W* is the sample mass, *D* = [exp (−λ *t_d_*)] is the decay factor, and *C* = ([1 − exp (−λ *t_m_*)]/λ *t_m_*) is the counting factor, *λ* decay constant, *t_c_*, *t_d_* and *t_m_* counting, decay and measurement times, respectively.

To check the accuracy of the analytical method, the parameter of the U-score test was determined. It was calculated according to the following equation;

$$U_{\mathrm{score}}=\frac{\left|X_{\mathrm{Lab}}-X_{\mathrm{Ref}}\right|}{\sqrt{\mu_{\mathrm{Lab}}^{2}+\sigma_{\mathrm{Ref}}^{2}}}$$

X_Lab_, μ_Lab_, X_Ref_ and σ_Ref_ refer to the laboratory results, the standard deviation, and the recommended and standard uncertainties, respectively. The laboratory performance was evaluated as satisfactory if U_score_ ≤ 1, and unsatisfactory for U_score_ < 1; the result and certified value were not in agreement [29].

#### 2.6.2. Determination of Mineral Elements by ICP-OES Technique

Determination of the mineral elements by the ICP-OES technique was performed by putting three samples of each collected charge weighing about 500 mg that were digested following the protocol previously adopted by the authors [30,31] after the full dissolution of the sample and allowing the solution to be homogenous by settling.

### 2.7. In Silico Studies

#### 2.7.1. Molecular Docking Validation Studies using Re-Docking and Superimposition

Validation of the docking experiments was achieved by comparing the alignments of the most stable docking poses of the lead compound together with the lead conformer co-crystallized with the respective enzyme from pdb. The value of RMSD (Root Mean Square Deviation) was determined to confirm the validity of the docking experiment and to show the ability to predict the binding affinity of new ligands.

#### 2.7.2. Molecular Docking

Molecular docking was performed for previously identified compounds by HPLC-UV-MS analysis conducted on Algerian *T. polium* aerial parts extract [14] using Discovery Studio 4.5 (Accelrys Inc., San Diego, CA, USA) with C-docker protocol. This was carried on human *α*-amylase (HA) (PDB ID 3BAY; 1.99 Å), 5-lipoxygenase (5-LOX) (PDB ID 6N2W, 2.71 Å) and acetylcholine esterase (PDB ID: 4EY7; 2.35 Å) obtained from the protein data bank (www.pdb.org) following what was previously reported [32,33]. This was compared with the reference drugs, namely acarbose, nordihydroguaiaretic acid and donepezil, which are co-crystalized with the previously mentioned enzymes, respectively. This was briefly performed in several steps, starting with the preparation of the structure of each enzyme by removing water molecules, followed by the addition of hydrogen atoms and then cleaning the protein structure from unwanted interactions. CHARMm was used as the forcefield, whereas MMFF94 was consumed for the calculation of partial charge calculation that was consequently accompanied by minimizing the added hydrogen in 2000 steps. Determination of the binding center was accomplished depending on the data reported approaching the catalytic domain of the targeted protein and the sphere site for the co-crystalized ligand. ChemDraw 13.0 was utilized to draw the 2D structures of the compounds that subsequently kept as pdb files. The default protocol for ligand preparation was adopted for the preparation of the 3D structures of the compounds for docking experiments. Molecular docking was then performed for the prepared structure inside the binding site spheres of the energy-minimized protein where the CHARMm force field was selected and the C-docker binding energy (ΔG) was calculated using a distance-dependent dielectric implicit solvation model for the best docking poses. Calculation of (ΔG) in Kcal/mol was performed by employing the following equation:

Δ*G*_binding_ = E_complex_ − (E_protein_ + E _ligand_) where;

Δ*G*_binding_: The ligand–protein interaction binding energy,

E_complex_: The potential energy for the complex of protein bound with the ligand,

E_protein:_ The protein potential energy alone

E_ligand_: The ligand potential energy alone

#### 2.7.3. ADMET and Drug Likeness Prediction

ADMET (absorption, distribution, metabolism, excretion and toxicity) determination for the prediction of the pharmacokinetic, pharmacodynamic and toxicity properties and drug likeness was performed on previously identified compounds by HPLC-UV-MS analysis, conducted on Algerian *T. polium* aerial parts extract using Biovia Discovery Studio software (Accelrys Inc., San Diego, CA, USA). Plasma protein binding prediction (PPB), human intestinal absorption, aqueous solubility, blood–brain barrier penetration (BBB), and cytochrome P450 (2D6) and hepatotoxicity level were chosen as ADMET parameters. However, ALogP, molecular weight (M.W.), number of hydrogen bond acceptors (HBA), number of hydrogen bonds donor (HBD), number of rings, number of aromatic rings and number of rotatable bonds were selected as drug likeness descriptors [34].

### 2.8. Statistical Analysis

Statistical analysis for in vitro experiments was performed by taking the means ± SD (n = 3) of three parallel measurements. For in vivo experiments, it was performed using student *t*-test and one way analysis (ANOVA), and data were expressed as mean ± SD. Graphs were constructed by GraphPad Prism version 5 software (GraphPad Software, Inc. La Jolla, CA, USA)

## 3. Results

### 3.1. Determination of the Biological Activity of T. polium Hydroalcoholic Extract

#### 3.1.1. Evaluation of Possible Antioxidant Activity In Vitro

The antioxidant activity of *T. polium* hydroalcoholic extract was estimated using DPPH: DPPH, GOR and *β* carotene assays. The results presented in Table 1 showed that *T. polium* hydroalcoholic extract showed moderate activity in galvinoxyl with IC_50_ of 21.82 μg/mL; it exhibited poor activity in *β* carotene assay, displaying an IC_50_ value of 165.41 μg/mL compared to the standards, butylated hydroxyanisole (BHA) and tert-butyl-1-hydroxytoluene (BHT). In contrast, it showed a significant antioxidant potential in DPPH assay with IC_50_ equals 8.68 μg/mL approaching that of BHA (IC_50_ = 6.14 μg/mL) and exceeding that of BHT (IC_50_ = 12.99) and vitamin E (α-Tocophérol (IC_50_ = 15.81).

#### 3.1.2. Evaluation of Possible Anti-Hyperglycemic Activity In Vitro

**Figure 1:**
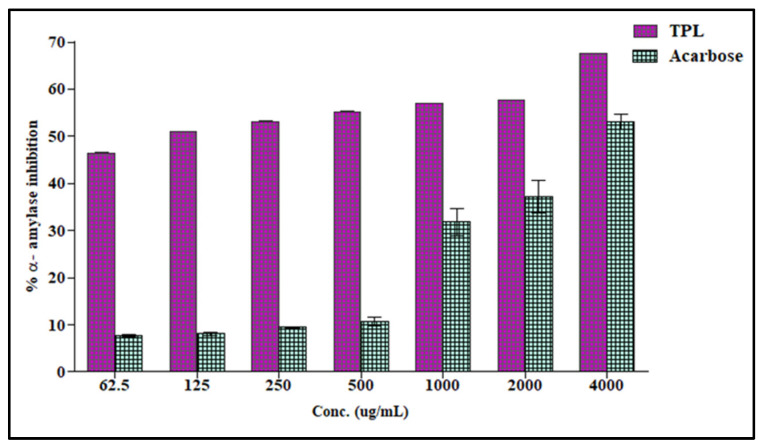
α-Amylase inhibitory activity of various concentrations (μg/mL) of *T. polium* hydroalcoholic extract (TPL) vs. acarbose; Values were expressed as means ± SD (n = 3) for three parallel measurements.

The anti-hyperglycaemic activity of *T. polium* hydroalcoholic extract was evaluated in vitro using the α-amylase inhibitory assay. The results illustrated in Figure 1 showed that *T. polium* hydroalcoholic extract showed a pronounced anti-hyperglycemic activity at the different assessed concentrations with IC_50_ of 111.68 µg/mL and thus exerted superior activity compared to acarbose, the standard drug (IC_50_ = 3650.93 µg/mL).

#### 3.1.3. Evaluation of Possible Anti-Alzheimer Activity In Vitro

**Figure 2:**
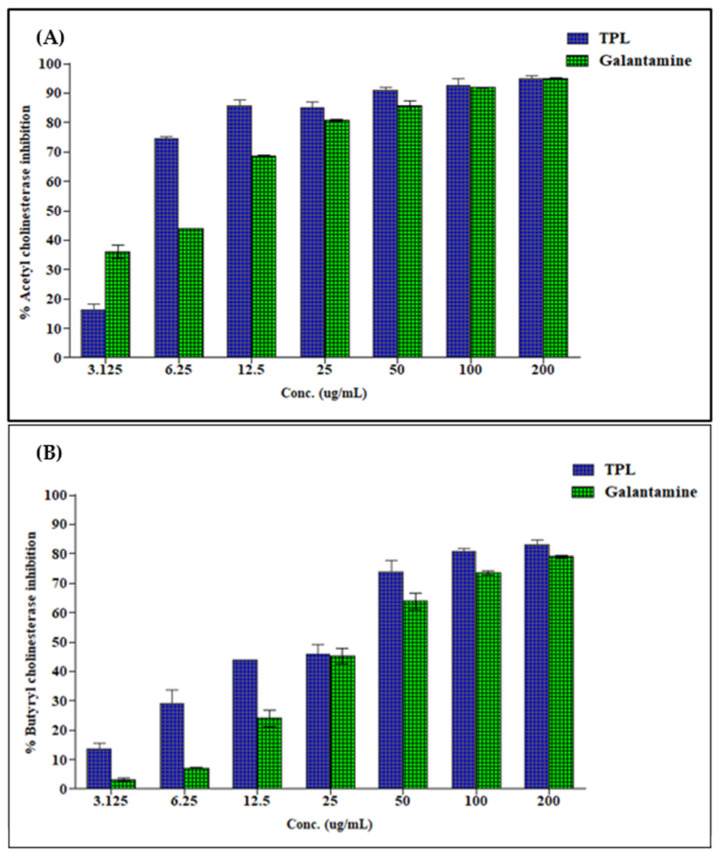
Acetylcholinesterase (**A**) and butyrylcholinesterase (**B**) inhibitory activity of various concentrations (μg/mL) of *T. polium* hydroalcoholic extract (TPL) vs. Galantamine: Values were expressed as means ± SD (n = 3) for three parallel measurements.

Herein, *T. polium* hydroalcoholic extract was evaluated in vitro for its inhibitory activity against AchE and BchE at different concentrations. Results illustrated in Figure 2 showed that *T. polium* showed excellent activity against BChE and AchE with IC_50_ values of 28.69 and 4.93 μg/mL, respectively, showing values superior to that exhibited by the standard galantamine with IC_50_ values of 34.75 and 6.27 μg/mL, for BchE and AChE, respectively.

#### 3.1.4. Evaluation of Possible Anti-Inflammatory Activity In Vitro

The anti-inflammatory activity of *T. polium* hydroalcoholic extract was evaluated in vitro using a BSA denaturation assay. Table 2 shows that the plant extract exhibited a strong anti-inflammatory effect with BSA denaturation inhibitory potential estimated by 97.53% at 2 mg/mL. This was closer to the inhibitory potential exerted by the standard drug, diclofenac sodium, which showed 100% inhibition at 2 mg/mL.

#### 3.1.5. Evaluation of the Anti-Inflammatory Activity In Vivo

Oral administration of *Teucrium polium* L. aerial parts hydroalcoholic extract to mice with carrageenan-induced hind paw edema produced significant (*p* < 0.001) anti-inflammatory activity at the tested doses. The present study showed that the hydroalcoholic extract of *T. polium* L. (10%) significantly reduced carrageenan-induced paw edema compared to the reference drug, diclofenac sodium (10 mg/kg). A gradual decrease in edema paw weight to 37.5% was observed compared to the standard drug diclofenac sodium, which exhibited 52.97% inhibition, as illustrated in Table 3. Thus, the obtained results confirmed the effect of the extract as an anti-inflammatory agent that could be attributed to the synergistic action of all of its biologically active phenolic compounds

### 3.2. Functional Phenolic Substances Predominating in Teucrium polium L. Aerial Parts

**Figure 3:**
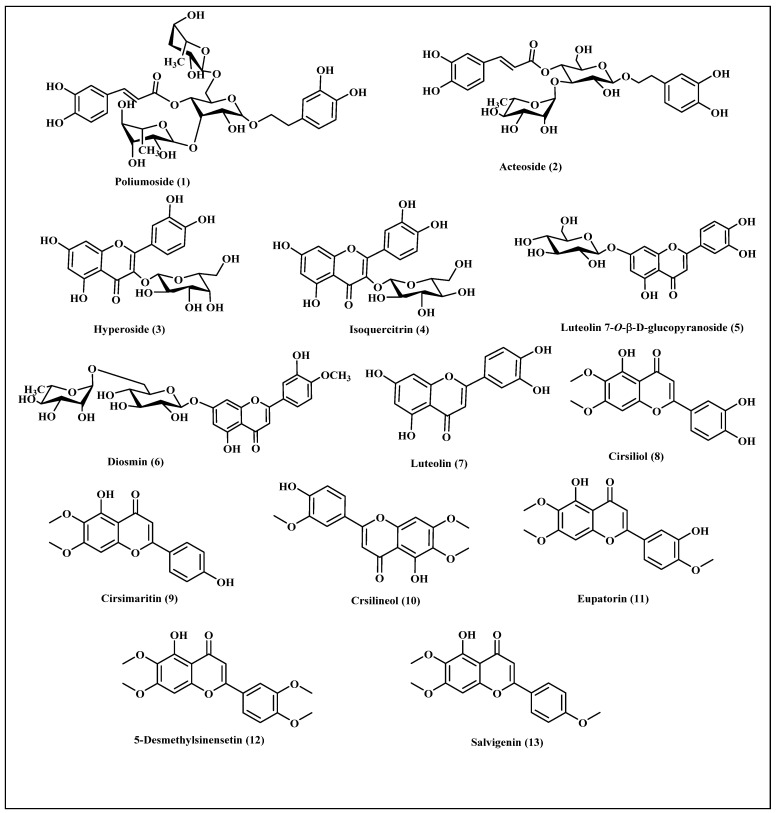
Scheme showing compounds previously identified from Algerian *Teucrium polium* aerial parts [14].

The extract of *T. polium* L. aerial parts is highly rich in phenolic compounds. Previous HPLC-UV-MS analysis conducted on the extract of Algerian *Teucrium polium* aerial parts revealed the existence of many phenolic compounds belonging mainly to flavonoids and caffeic acid derivatives. These include poliumoside **(1)**, acteoside **(2)**, hyperoside **(3)**, isoquercitrin **(4)**, luteolin 7-O-*β*-D-glucopyranoside **(5)**, diosmin **(6)**, luteolin **(7)**, cirsiliol **(8)**, cirsimaritin **(9)**, cirsilineol **(10)**, eupatorin **(11)**, 5-desmethylsinensetin **(12)** and salvigenin **(13)** [14]. A scheme showing compounds previously identified from Algerian *Teucrium polium* aerial parts is illustrated in Figure 3.

### 3.3. Determination of the Total Content of Phenol and Flavonoids

The total content of phenol and flavonoids was determined using the Folin–Ciocalteu method, where results revealed that *Teucrium polium* aerial parts were rich in polyphenols with 36.35 ± 0.294 μg GAE/mg and in flavonoids with 24.30 ± 0.44 μg QE/mg. The polyphenol content was expressed in mg of gallic acid, equivalent per gram of dry weight; the flavonoid content was expressed in mg of quercetin equivalent per gram of dry weight.

### 3.4. Determination of Mineral Elements

The present study evaluated the mean values of elemental mineral concentrations in *Teucrium polium* L. growing in Algeria. This was performed using the instrumental neutron activation analysis method, and the results are demonstrated in Table 4. Nineteen minerals comprising macro- and microelements, namely Ba, Br, Co, Cr, Cs, Eu, Fe, K, Mo, Na, Sb, Sc, Sr, U and Zn, were determined by both techniques, which were INAA and ICP-OES. The data presented in this experiment showed that six micronutrient elements “essential chemical” were quantified in this plant analysis; their levels are arranged as follows; K > Fe > Na > Zn > Cr > Co. However, two potential toxic elements were found, Br and Sb, in addition to seven other elements.

### 3.5. In Silico Studies

#### 3.5.1. Molecular Docking Validation Studies using Re-Docking and Superimposition

Validation experiments revealed an acceptable alignment between the docking pose of the lead compounds that showed the best fitting with the co-crystallized lead conformers. They displayed RMSD values of 1.18, 1.74, and 2.50 Å for human α-amylase, 5-lipoxygenase and acetylcholine esterase, respectively (Figure S1).

#### 3.5.2. Molecular Docking

Aiming to consolidate the results obtained from the in vitro studies and provide a mechanistic postulation for the pronounced activity of *Teucrium polium*, molecular docking was performed for previously identified compounds in the extract of Algerian *T. polium* aerial parts on human *α*-amylase, 5-lipoxygenase and acetylcholine esterase (Table 5).

**Figure 4:**
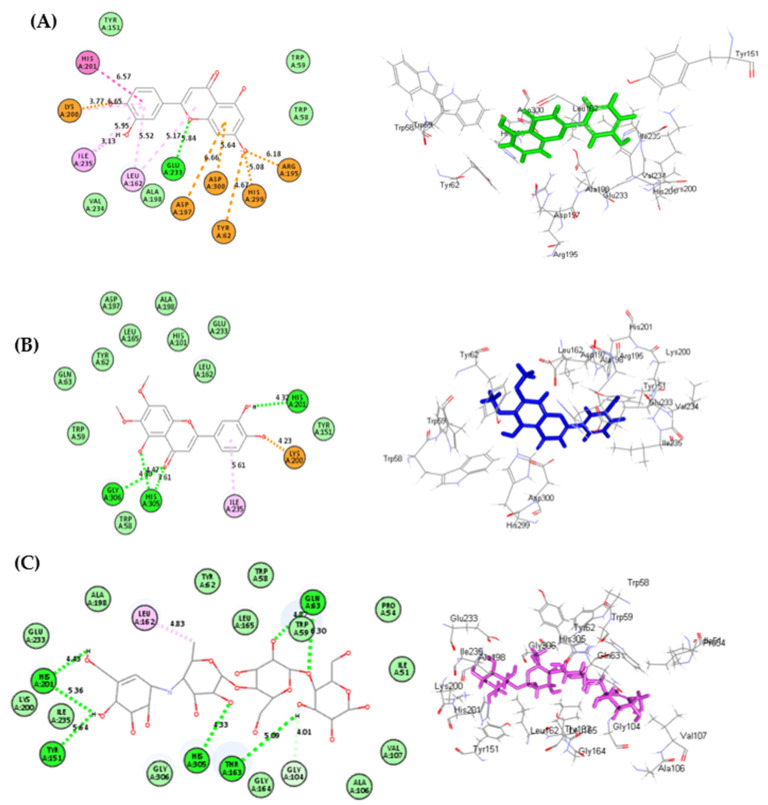
The 2D and 3D binding modes of luteolin (**A**), cirsiliol (**B**) and acarbose (**C**) inside the active site of human *α*-amylase; heavy green dotted bond, H-bonds; heavy pink dotted bond, π-π bonds; light green dotted bond, C-H bonds; light pink dotted bond, π-alkyl bonds; dotted orange bonds, salt bridge interactions.

Regarding human *α*-amylase, luteolin **(7)** showed the best fitting, followed by cirsiliol **(8)**, displaying ∆G of −47.89 and −37.11 kcal/mol, respectively, approaching that of acarbose, which showed ∆G of −75.26 kcal/mol. Luteolin forms one conventional H-bond with Glu233; three π-anion bonds with Asp197, Asp300 and Tyr62; one π-π T-shaped bond with His201; four π-alkyl bonds with Lys200, Leu162, Ile235 in addition to three salt bridge interactions with His299, Arg195 and Lys200 (Figure 4A). However, cirsiliol forms four conventional H-bonds with Gly306, His201 and Tyr151; one π-alkyl bond with Ile235 and one salt bridge interactions with Lys200 (Figure 4B). Acarbose (Co-crystalized ligand) forms seven conventional H-bonds with His201, Tyr151, His305, Thr163, Gln63 and Trp59; one alkyl interaction with Leu162 in addition to one C-H bond with Gly104 (Figure 4C). Additionally, most of the examined compounds showed a promising degree of interaction with the binding sites, such as acteoside **(2)**, hyperoside **(3)**, isoquercitrin **(4)**, luteolin 7-O-*β*-D-glucopyranoside **(5)**, cirsimaritin **(9)**, cirsilineol **(10)**, eupatorin **(11)**, 5-desmethylsinensetin **(12)** and salvigenin **(13)**, owing to the formation of multiple bonds with the amino acid existing at the binding center such as H-bonds, π-π, π-alkyl and slat bridge interactions. The 2D binding mode of major compounds identified from Algerian *Teucrium polium* aerial parts inside the active site of human *α*-amylase was illustrated in Figure S2.

**Figure 5:**
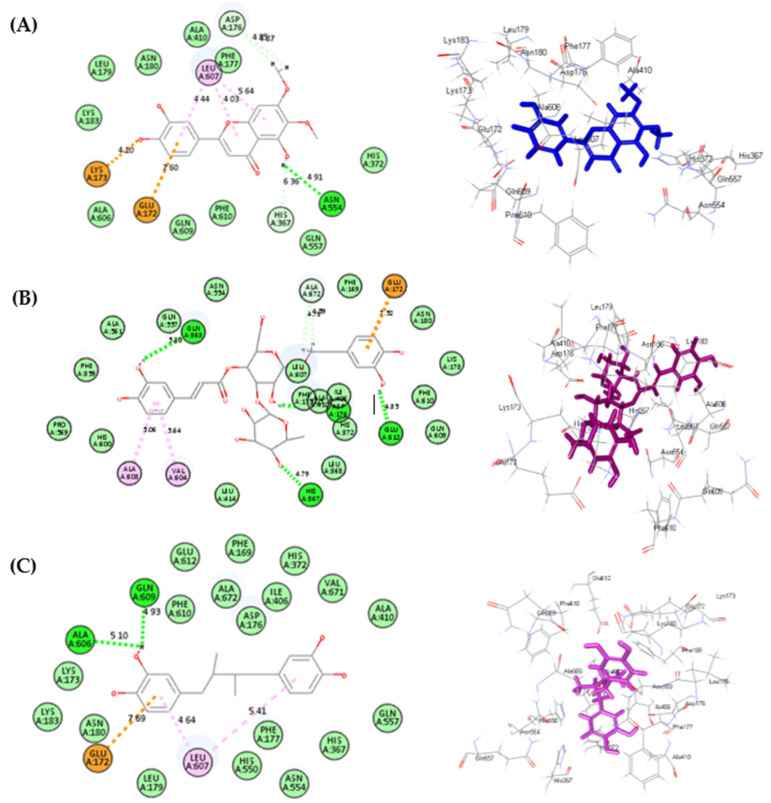
2D and 3D binding modes of cirsiliol (**A**), acetoside (**B**) and nordihydroguaiaretic acid (**C**) inside the active site of 5-lipoxygenase, heavy green dotted bond, H-bonds; heavy pink dotted bond, π-π bonds; light green dotted bond, C-H bonds; light pink dotted bond, π-alkyl bonds; dotted orange bonds, π-anion interaction.

Concerning 5-lipoxygenase, cirsiliol **(8)** followed by acteoside **(2)** revealed the most firm fitting with the active sites displaying ∆G of −40.04 and −38.49 kcal/mol, respectively, approaching that of nordihydroguaiaretic acid with ∆G of −43.37 kcal/mol. Cirsiliol forms one H-bond with Asn554; one π–anion interaction with Glu172; one attractive charge interaction with Lys173; three π–alkyl interactions with Leu607; three C-H bonds with Asp176 and His367 (Figure 5A). However, acteoside forms two π–alkyl interactions with Ala508 and Val504; two C-H bonds with Ala572; one π–anion interaction with Glu172 and four H-bonds with Gln963, Glu612 and His967 (Figure 5B). Nordihydroguaiaretic acid (Co-crystalized ligand) forms two H-bonds with Ala606 and Gln609, one π–anion interaction with Glu172, and two π–alkyl interactions with Leu607 (Figure 5C). In addition, all other major tested compounds showed a pronounced fitting within the binding site that is mainly attributed to the formation of plethora of bonds with the amino acid residues where the 2D binding mode of the major compounds identified from Algerian *Teucrium polium* aerial parts inside the active site of 5-lipoxygenase was illustrated in Figure S3.

**Figure 6:**
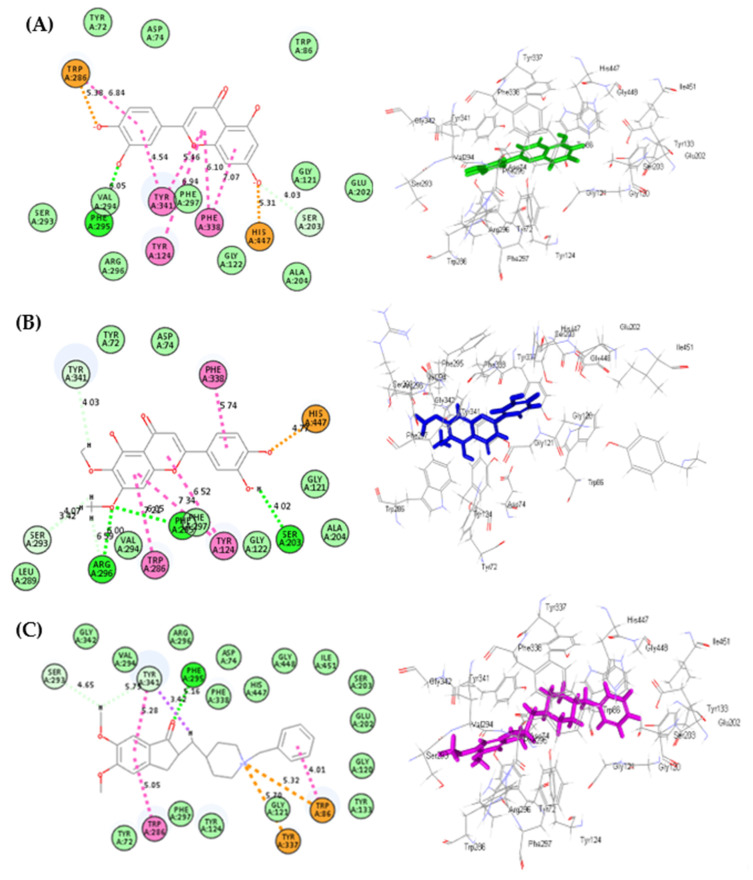
The 2D and 3D binding modes of luteolin (**A**), cirsiliol (**B**) and donepezil (**C**) inside the active site of acetylcholine esterase; heavy green dotted bond, H-bonds; heavy pink dotted bond, π-π bonds; light green dotted bond, C-H bonds; light pink dotted bond, π-alkyl bonds; purple dotted bond, π-δ bond; dotted orange bonds, π-cation interaction.

In addition, luteolin **(7)** showed the best fitting within acetylcholinesterase active sites, followed by cirsiliol **(8)**, displaying ∆G of −49.55 and −41.79 kcal/mol, respectively, showing superior activity compared to donepezil, which showed ∆G of −25.89 kcal/mol. Luteolin forms one H-bond with Phe295; one C-H bond with Ser203; one salt bridge with His447; one π-anion with Trp286 in addition to six π–π interactions with Tyr341, Tyr124 and Phe338 (Figure 6A). Moreover, cirsiliol forms one salt bridge with His447; four π–π interactions with Trp286, Tyr124 and Phe338; two H-bonds with Arg296, Phe225 and Ser203, in addition to two C-H bonds with Ser293 and Tyr341(Figure 6B). However, donepezil, the co-crystalized ligand, forms two π–cation interactions with Trp86 and Tyr337; three π-π bonds with Tyr341, Trp286 and Trp86; one H-bond with Phe295; two C-H bonds with Ser293 and Tyr341 in addition to one π-δ bond with Tyr341 (Figure 6C). Thus, molecular docking results further ascertained the in vitro results and highlighted that the secondary metabolites of *Teucrium polium* target many crucial proteins. Consequently, *Teucrium polium* showed pronounced biological activities. Concerning the rest of the major compounds, they exert pronounced activity within the binding site, where the 2D binding mode of all major compounds identified from Algerian *Teucrium polium* aerial parts inside the active site of acetylcholine esterase is illustrated in Figure S4.

#### 3.5.3. ADMET and Drug Likeness Prediction

**Figure 7:**
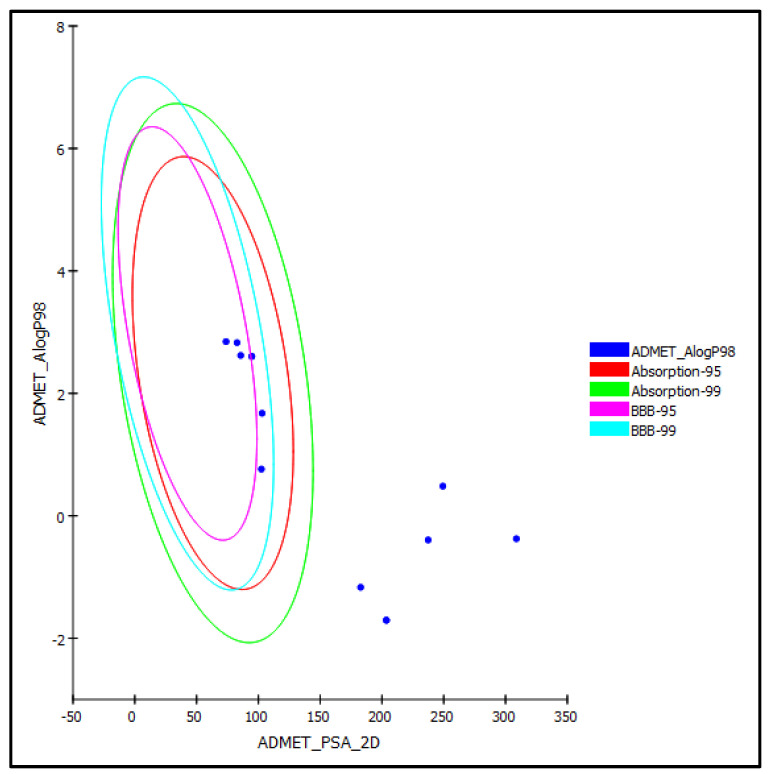
ADMET Plot of Algerian *T. polium* aerial parts phenolic compounds displaying 95% and 99% confidence limit ellipses with respect to the human intestinal absorption and the blood–brain barrier (BBB) models.

ADMET (absorption, distribution, metabolism, excretion and toxicity) determination for the prediction of the pharmacokinetic, pharmacodynamic and toxicity properties and drug likeness was performed on previously identified phenolic compounds on Algerian *T. polium* aerial parts extract. Results illustrated in Table 6 and Figure 7 revealed that compounds **(7–13)** showed good human intestinal absorption consequently allocated in the 99% absorption ellipse whereas compounds **(1–6)** showed very low human intestinal absorption and concomitantly lie outside 99% absorption ellipse, as revealed in Figure 7. Regarding solubility level, most of the tested compounds showed good and optimal solubility except poliumoside **(1)**, acteoside **(2)** and diosmin **(6)** that showed low to very low but possible solubility. In addition, compounds **(1–6)** showed undefined, penetration via BBB and hence placed outside the 99% BBB confidence eclipse in contrast to compounds **(7–13)** that showed medium to low penetration via BBB lying inside 95 and 99% BBB confidence eclipse. Additionally, compounds **(1–6)** showed less than 90% plasma protein binding (PPB) in contrast to compounds **(7–13)** that revealed more than 90% PPB. In addition, all of the examined compounds are non-CYP2D6 inhibitors. Unfortunately, most of the compounds showed certain hepatic toxicity except compounds **(1–4)** that showed no toxicity. Additionally, results illustrated in Table 7 reflected the suitability of most of the examined compounds for their incorporation in different dosage forms, particularly those that revealed promising biological activity as evidenced from their molecular weight (M.W.), number of hydrogen bond acceptors (HBA), number of hydrogen bond donors (HBD), number of rings, number of aromatic rings and number of rotatable bonds.

## 4. Discussion

*Teucrium polium* L. belonging to the family Lamiaceae is widely used in traditional medicine to treat hypertension, diabetes, or as a wound-healing agent [13]. Results illustrated in this study showed that *Teucrium polium* aerial parts are rich in polyphenols with 36.35 ± 0.294 μg GAE/mg and in flavonoids with 24.30 ± 0.44 μg QE/mg. Variation in geographical origin undoubtedly influences the total content of phenol and flavonoids in the same species [35]. The extract of *T. polium* growing in Morocco demonstrated a higher polyphenol content estimated by 95.53 ± 1.65 mg GAE/g in addition to a higher flavonoid content reported to be 101.9 ± 1.97 mg RE/g (Rutin equivalent per gram of dry weight) [36]. Furthermore, the extract of Tunisian *T. polium* revealed a lower flavonoid content that was estimated to be 2.67 ± 0.05 mg RE/g [37]. In addition, *T. polium* L. aerial parts are highly rich in phenolic compounds, as evidenced by HPLC-UV-MS analysis previously conducted on the extract of Algerian *Teucrium polium* aerial parts that revealed the existence of many phenolic compounds belonging mainly to flavonoids and caffeic acid derivatives [14].

Additionally, the evaluation of elemental mineral concentrations in *Teucrium polium* L. growing in Algeria was conducted for the first time. Mineral elements, particularly the micro and macro-nutrients are essential to various human metabolic processes and significantly contribute to human health [38]. The essential elements of Na, Fe and K were detected as the highest level among the other elements, where K showed the highest level, estimated at 7619 mg/kg, followed by Fe (984 mg/kg) and then Na (803 mg/kg). In addition, the concentration of essential elements acting as micronutrients, such as Zinc, Chromium and Cobalt, ranged from 24 to 0.48 mg/kg. Although two potential toxic elements were detected, their levels lie below the tolerance limits compared with the recommended values (RDA) [39].

Moreover, *Teucrium polium* L. showed pronounced antioxidant, antihyperglycaemic, anti-Alzheimer activity and anti-inflammatory activity exceeding the used standards. This is mainly attributed to its richness with phenolic compounds, particularly flavonoids highlighted by the detected total flavonoid contents and previously isolated compounds. Flavonoids and phenolics are greatly popular as the largest phytochemical entities with pronounced antioxidant properties from plants [40,41]. Regarding the antioxidant activity, the obtained results follow what was previously reported by Sharififar et al. [42], which reported the antioxidant potential of the Iranian *T. polium* hydroalcoholic extract owing to its free radicle scavenging properties. However, herein, the reported results from the Algerian species showed better antioxidant potential using DPPH assay from the previously reported the Iranian species that showed the IC_50_ value of 20.1 ± 1.7 µg/mL. The variation in the obtained results reflected the effect of geographical origin in altering to some extent the biological activity that is, in turn, influenced by the secondary metabolites. Moreover, flavonoids significantly prohibit oxidative stress-related diseases via scavenging of reactive oxygen species (ROS) directly by different mechanisms comprising antioxidant enzymes stimulation, inhibition of nitric oxide-induced oxidative stress in addition to metal-chelating activity [43], whereas scavenging of free radicals is considered the most important mode of antioxidant action of flavonoids as the polyphenol groups interrupt the free radical chain reaction [44]. Regarding the structure–activity relationship, for efficient radical scavenging behavior, the critical structural features are manifested by the presence of an ortho-dihydroxy structure in ring B, which is critical for electron delocalization; the existence of a 2,3 double bond in conjugation with a 4-keto function that allows the electron delocalization from the B ring in addition to the presence of hydroxyl groups at positions 3 and 5 provides hydrogen bonding to the keto groups that are present in most of the identified compounds in [44]. It is noteworthy to highlight that the antioxidant potential of phenolic compounds and flavonoids is greatly influenced by functional group arrangement, substitution, configuration, the number of hydroxyl groups that, in turn, affect metal ion chelation ability and radical scavenging activity [45,46].

Furthermore, the pronounced antihyperglycaemic activity of *Teucrium polium* aerial parts evidenced by an in vitro study and further supported by in silico studies greatly relied upon the richness of *Teucrium polium* L. with flavonoids. It is worth highlighting that *T. polium* hydroalcoholic extract showed one hundred and thirty times higher activity than acarbose, which could be attributed to the richness of the plant with various phytochemicals such as flavonoids, tannins, and saponins [47]. Previous studies on α-amylase inhibitors identified from medicinal herbs revealed that several potent inhibitors belong to the flavonoid class [32]. The obtained results further consolidated what was previously reported by Dastjerdi et al. [48], showing that Iranian *T. polium* possesses a very good activity in α-amylase inhibitory assay with IC_50_ values of 3.01 and 1.64 mg/mL for the dichloromethane extract and with ethyl acetate extracts, respectively. Flavonoids elicited an antihyperglycemic effect via suppressing α-glucosidase and α-amylase activities, attenuating insulin resistance and promoting pancreatic cell proliferation [5,49]. Moreover, many studies further supported the results of in silico studies, where luteolin revealed a potent antihyperglycemic effect via inhibition of α-amylase [50]. In addition, luteolin showed an improved insulin action by direct activation of the PPAR pathway, by acting at the insulin signaling pathway, and GLUT4 expression in addition to the up-regulation of synaptic proteins expression and improving endothelial insulin resistance responsible for inflammation [51]. Flavonoid antihyperglycemic activity may also elicit α-glucosidase inhibition. This inhibition was greatly influenced by the hydroxylation and galloylation of flavonoids that improved the inhibitory activity. On the contrary, the glycosylation of the hydroxyl group and hydrogenation of the C2=C3 double bond on flavonoids weaken the inhibition; however, caffeoylquinic acids showed strong prohibition of α-glucosidase [52].

Concerning the anti-Alzheimer activity of *Teucrium polium* aerial parts, it can be concluded that *T. polium* could be used in the alleviation of Alzheimer’s evidenced by its promising BChE and AChE inhibitory potential. This activity is perhaps due to its richness in polyphenols since the members of Lamiaceae were found to be rich in phenolic acids as active constituents that significantly contribute to their neuroprotective properties [53]. Moreover, a study by Valdimir-Knežević et al. [54] on the ethanol extract of several Lamiaceae cultivated in Croatia showed that *T. polium* was among the most potent plant extracts, with significant AchE inhibitory rates exceeding 75% at 1 mg/mL concentration. Alzheimer’s disease is a form of dementia that is characterized by the presence of senile plaques, neurofibrillary tangles with concomitant synaptic loss and neuronal death leading to gradual memory loss, a decrease in language skills in addition to cognitive impairments [55]. The neurotransmitter acetylcholine has a crucial role in learning process and memory in the hippocampus. Two enzymes, acetylcholinesterase (AChE) and butyrylcholinesterase (BChE), are involved in the of acetylcholine hydrolysis, decreasing its level in Alzheimer’s disease, and thus, prohibition of both enzymes is a well-established strategy for the alleviation of Alzheimer’s disease [56]. Flavonoids are promising natural products with neuroprotective potential that prevent or slow the progression of Alzheimer disease via the inhibition of key enzymes such as AChE, BChE and BACE-1 (Beta-site APP Cleaving Enzyme-1) [57]. Moreover, Shimmyo et al. showed that flavonols and flavones are capable of inhibiting BACE-1 where the presence of OH groups of C3′ and C4′ stabilized the binding poses of flavonoids within the BACE-1 active center via hydrogen bond formation. Additionally, the existence of OH at C3 interacted in a direct manner with the Asp catalytic residue, causing a notable enhancement of the BACE-1 inhibitory activity [58]. Moreover, previous studies reported the effectiveness of cirsiliol as a neuroprotective agent and sedative acting as CNS depressants at the GABA chloride channel and/or at glutamate binding sites that further supported the in silico studies [59].

Concerning the anti-inflammatory activity of *Teucrium polium* aerial parts, its pronounced activity evidenced by BSA denaturation inhibitory potential estimated as 97.53% at 2 mg/mL mainly relied upon its richness with flavonoids. It is worth highlighting that water/ethanol is an effective solvent in extracting phytochemicals from the plant as it combines polar and medium polarity properties [60]. Thus, it appears that the anti-inflammatory effect of the extract may be due to the presence of flavonoids and phenolic compounds in the plant [54,61]. Cirsiliol has been reported to possess a potent and selective 5-lipoxygenase inhibitory potential that further consolidated the in silico studies [62]. Several mechanisms of action have been suggested in an effort to interpret the mode of action of flavonoids, such as their antioxidant potential, their ability to modulate the production of proinflammatory cytokines and the expression of genes [63,64]. Thus, flavonoids hinder the inflammation process by a reduction in cytokines and inflammatory markers expression and via interaction with proteins incorporated in the incidence of inflammation. Additionally, they modulate arachidonic acid (AA)-metabolizing enzymes activity, such as COX, phospholipase A2 (PLA2), lipoxygenase (LOX), as well as the NO-producing enzyme nitric oxide synthase (NOS) and thus reduce the production of AA, PG, leukotriene, and NO, which are critical mediators of inflammation accounting for cellular mechanisms of anti-inflammation [65]. Luteolin was previously shown to inhibit the rat renal medulla COX with an IC_50_ of 100–130 µM, whereas other flavonoids exhibited a significant inhibition of LOX where the reduction in the C-2, 3-double bond and glycosylation reduced the flavonoids inhibitory activities [63]. Thus, *T. polium* can act as a good candidate for alleviating many health-debilitating problems and can be highly beneficial in the pharmaceutical industry and medical research.

## 5. Conclusions

Herein, *Teucrium polium* L. was evaluated for its antioxidant, antihyperglycaemic, anti-Alzheimer activity and anti-inflammatory activity by different assays in vitro and in vivo and then confirmed by in silico studies for the first time. In addition, the minerals were investigated by two techniques, namely instrumental neutron activation analysis (INAA) and inductively coupled plasma-optical emission spectrometry (ICP-OES), for the first time as they are highly important, either micro or macronutrients, and essential for various human metabolic processes. Results showed that *Teucrium polium* L. is rich in minerals necessary for humans, such as calcium, potassium, iron, sodium, magnesium, manganese and zinc. In addition, it showed promising antioxidant, antihyperglycaemic, anti-Alzheimer activity and anti-inflammatory activity, which could be attributed to its phenolic compounds as confirmed by in silico studies. Thus, it can be concluded that *T. polium* can act as a good candidate for alleviating many health-debilitating problems and could be incorporated into various pharmaceutical preparations. However, additional in vivo and preclinical trials are highly recommended to ascertain the obtained results. Additionally, performing docking experiments to examine the inhibitory potential of the main compounds in Algerian *T. polium* extract versus butyrylcholinesterase and beta-secretase 1 enzyme is highly recommended.

## Figures and Tables

**Table 1:** In vitro antioxidant activity of *T. polium* hydroalcoholic extract (TPL) using DPPH, GOR and *β* carotene assays expressed in terms of IC_50_ (μg/mL).

Samples	DPPH	GOR	*β* Carotene
TPL	8.68 ± 0.33	21.82 ± 0.19	165.41 ± 1.57
BHT	12.99 ± 0.41	3.32 ± 0,18	0.91 ± 0.01
BHA	6.14 ± 0.41	5.38 ± 0,06	1.05 ± 0.03
Vitamin E	15.81 ± 0.54	-	-

Values were expressed as means ± SD (n = 3) for three parallel measurements.

**Table 2:** In vitro anti-inflammatory activity of various concentrations (μg/mL) of *T. polium* hydroalcoholic extract (TPL) using BSA denaturation assay versus diclofenac sodium.

Concentration (μg/mL)	TPL	Diclofenac Sodium
2000	97.53 ± 0.47	100 ± 0.18
1000	95.76 ± 0.18	92 ± 0.15
500	91.03 ± 0.28	61 ± 0.15
250	80.35 ± 3.18	37 ± 0.18

**Table 3:** In vivo anti-inflammatory activity of *T. polium* hydroalcoholic extract (TPL) using carrageenan-induced hind paw edema versus diclofenac sodium.

Samples	Average Paw Weight (g)		% Oedema	% Oedema Reduction
	Left	Right		
Control “Physiological water”	0.109 ± 0.006	0.076 ± 0.004	43.42%	0%
Standard “Diclofenac sodium”	0.171 ± 0.009	0.142 ± 0.001	20.42%	52.97%
TPL	0.178 ± 0.001	0.140 ± 0.001	27.14%	37.49%

**Table 4:** Results of chemical element’s mass fractions (mg/kg on dry mass basis) determined in *Teucrium polium* L. by INAA and ICP-OES techniques.

Elements		*Teucrium polium* L.	The Used Technique
1	Ba	5.10 ± 0.67	INAA
2	Br	18.86 ± 11.56	INAA
3	Ca	18267 ± 386	ICP
4	Cl	219.5 ± 3	INAA
5	Co	0.480 ± 0.041	INAA
6	Cr	2.29 ± 0.65	INAA; ICP
7	Cs	0.111 ± 0.023	INAA
8	Eu	0.040 ± 0.015	INAA
9	Fe	983.9 ± 111.9	INAA; ICP
10	K	7619 ± 2295	INAA
11	Mg	230.2 ± 2	INAA; ICP
12	Mn	39.3 ± 5	INAA
13	Mo	0.28 ± 0.101	INAA
14	Na	803 ± 41	INAA
15	Sb	0.050 ± 0.013	INAA
16	Sc	0.012 ± 0.001	INAA
17	Sr	138.03 ± 7.97	INAA
18	U	0.040 ± 0.013	INAA
19	Zn	24.22 ± 1.04	INAA; ICP

Values were expressed as means ± SD (n = 3) for triplicate analyses, multiple irradiations or different photo peaks.

**Table 5:** Free binding energies (kcal/mol) of the main identified compounds in the Algerian *T. polium* extract (TPL) used inside the active site of human *α*-amylase, 5-lipoxygenase and acetylcholine esterase using in silico studies.

Compounds	Human *α*-Amylase	5-Lipoxygenase	Acetylcholine Esterase
Poliumoside **(1)**	FD	−10.10	FD
Acteoside **(2)**	−35.50	−38.49	−18.48
Hyperoside **(3)**	−30.53	−19.43	−17.22
Isoquercitrin **(4)**	−33.79	−18.90	−13.99
Luteolin 7-O-*β*-D glucopyranoside **(5)**	−29.49	−17.17	−24.29
Diosmin **(6)**	83.64	−9.54	−0.43
Luteolin **(7)**	−47.89	−36.53	−49.55
Cirsiliol **(8)**	−37.11	−40.04	−41.79
Cirsimaritin **(9)**	−35.46	−15.93	−22.99
Cirsilineol **(10)**	−35.50	−18.27	−25.67
Eupatorin **(11)**	−25.95	−27.66	−34.66
5-Desmethylsinensetin **(12)**	−23.38	−20.78	−30.77
Salvigenin **(13)**	−23.56	−21.789	−30.08
Acarbose (Co-crystalized ligand)	−75.26	ND	ND
Nordihydroguaiaretic acid (NDGA) (Co-crystalized ligand)	ND	−43.37	ND
Donepezil (Co-crystalized ligand)	ND	ND	−25.89

FD: fail to dock; ND: not done.

**Table 6:** ADMET (absorption, distribution, metabolism, excretion, and toxicity) properties of phenolic compounds in Algerian *T. polium* aerial parts extract.

Compounds	Absorption Level	Solubility Level	BBB Level	PPB Level	CPY2D6	Hepatotoxic	PSA-2D	Alog p98
Poliumoside **(1)**	3	1	4	False	NI	NT	308.782	−0.375
Acteoside **(2)**	3	2	4	False	NI	NT	249.29	0.484
Hyperoside **(3)**	3	3	4	False	NI	NT	203.585	−1.706
Isoquercitrin **(4)**	3	3	4	False	NI	NT	203.585	−1.706
Luteolin 7-O-*β*-D glucopyranoside **(5)**	3	4	4	False	NI	Toxic	182.77	−1.168
Diosmin **(6)**	3	2	4	False	NI	Toxic	237.405	−0.395
Luteolin **(7)**	0	3	3	True	NI	Toxic	102.463	0.762
Cirsiliol **(8)**	0	3	3	True	NI	Toxic	103.022	1.674
Cirsimaritin **(9)**	0	3	3	True	NI	Toxic	85.722	2.619
Cirsilineol **(10)**	0	3	3	True	NI	Toxic	94.652	2.603
Eupatorin **(11)**	0	3	3	True	NI	Toxic	94.652	2.603
5-Desmethylsinensetin **(12)**	0	3	3	True	NI	Toxic	82.766	2.828
Salvigenin **(13)**	0	3	2	True	NI	Toxic	73.836	2.845

0, 1, 2, and 3 indicates good, moderate, low and very low absorption, respectively; 0, 1, 2, 3, 4, and 5 indicates extremely low, very low but possible, low, good, optimal, and too soluble, respectively; 0, 1, 2, 3, and 4 denote very high, high, medium, low, and undefined, penetration via BBB respectively. PBB, plasma protein binding; false = less than 90%, True = more than 90%; NI: Non-inhibitor; NT: Non-toxic.

**Table 7:** Drug-likeness predictions of phenolic compounds in Algerian *T. polium* aerial parts extract through using Biovia Discovery Studio software.

Compounds	ALogP	M.W	HBD	HBA	N° of Aromatic Rings	N° of Rings	N° of Rotatable Bonds
Poliumoside **(1)**	−0.375	770.728	11	19	2	5	13
Acteoside **(2)**	0.484	624.587	9	15	2	4	11
Hyperoside **(3)**	−1.706	462.36	6	12	2	4	4
Isoquercitrin **(4)**	−1.706	462.36	6	12	2	4	4
Luteolin 7-O-*β*-D glucopyranoside **(5)**	−1.168	446.361	5	11	2	4	4
Diosmin **(6)**	−0.395	608.545	8	15	2	5	7
Luteolin **(7)**	0.762	284.22	2	6	2	3	1
Cirsiliol **(8)**	1.674	329.281	2	7	2	3	3
Cirsimaritin **(9)**	2.619	314.289	2	6	2	3	3
Cirsilineol **(10)**	2.603	344.315	2	7	2	3	4
Eupatorin **(11)**	2.603	344.315	2	7	2	3	4
5-Desmethylsinensetin **(12)**	2.828	358.342	1	7	2	3	5
Salvigenin **(13)**	2.845	328.316	1	6	2	3	4

## Data Availability

Data are available in the manuscript.

## Supplementary Materials

The following supporting information can be downloaded at: https://www.mdpi.com/article/10.3390/life12101579/s1, Figure S1: Validation of the docking experiments for human α-amylase, (A), 5-lipoxygenase (B) and acetylcholine esterase, (C); Figure S2: 2D binding mode of compounds identified from Algerian *Teucrium polium* aerial parts inside the active site of human *α*-amylase; Figure S3: 2D binding mode of compounds identified from Algerian *Teucrium polium* aerial parts inside the active site of 5-lipoxygenase; Figure S4: 2D binding mode of compounds identified from Algerian *Teucrium polium* aerial parts inside the active site of acetylcholine esterase.

## Abbreviations

ACHE	acetylcholinesterase
BChE	butyrylcholinesterase
DPPH	2,2-diphenyl-1-picrylhydrazyl
GOR	galvinoxyl radical
INAA	instrumental neutron activation analysis
ICP-OES	inductively coupled plasma—optical emission spectrometry
