# Supplementary material for: Evaluation of Possible Antioxidant, Anti-Hyperglycaemic, Anti-Alzheimer and Anti-Inflammatory Effects of Teucrium polium Aerial Parts (Lamiaceae)
Source: Life (Basel). 2022 Oct 11;12(10):1579. doi: 10.3390/life12101579 (PMC9604868; doi:10.3390/life12101579)
Supplement: Supplementary file 1 [file life-12-01579-s001.zip › life-1938793-supplementary.pdf]

Evaluation of possible antioxidant, anti-hyperglycaemic, anti-Alzheimer and anti-inflammatory effects of *Teucrium polium* aerial parts (Lamiaceae)

Supplementary data

Figure S1: Validation of the docking experiments for human α-amylase, (**A**), 5-lipoxygenase (**B**) and acetylcholine esterase, (**C**).

Figure S2: 2D binding mode of compounds identified from Algerian *Teucrium polium* aerial parts inside the active site of human α-amylase

Figure S3: 2D binding mode of compounds identified from Algerian *Teucrium polium* aerial parts inside the active site of 5-lipoxygenase

Figure S4: 2D binding mode of compounds identified from Algerian *Teucrium polium* aerial parts inside the active site of acetylcholine esterase

Figure S1: Validation of the docking experiments for human α-amylase, (**A**), 5-lipoxygenase (**B**) and acetylcholine esterase, (**C**).

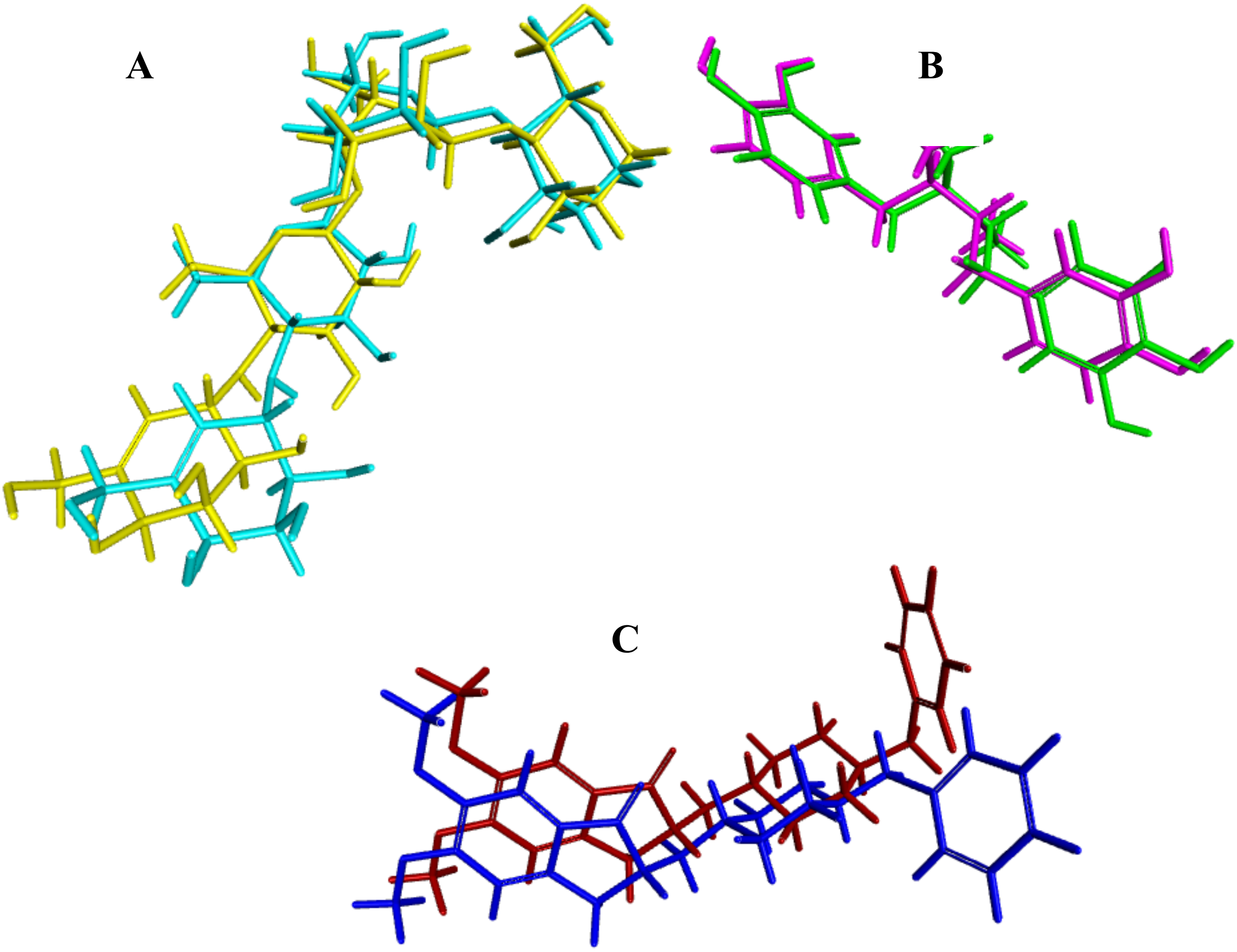

Figure S2: 2D binding mode of compounds identified from Algerian *Teucrium polium* aerial parts inside the active site of human α-amylase

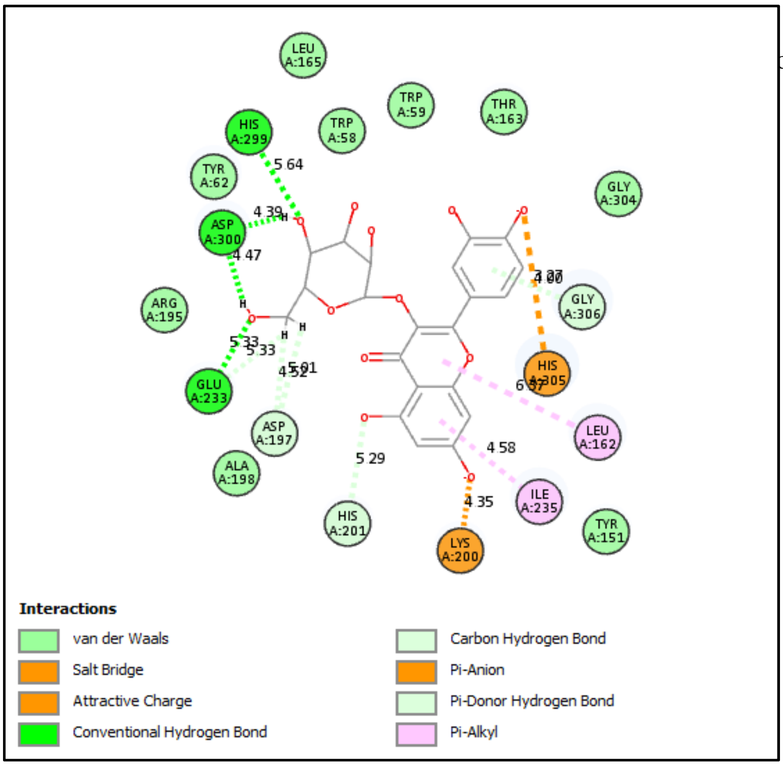

Hyperoside (3)

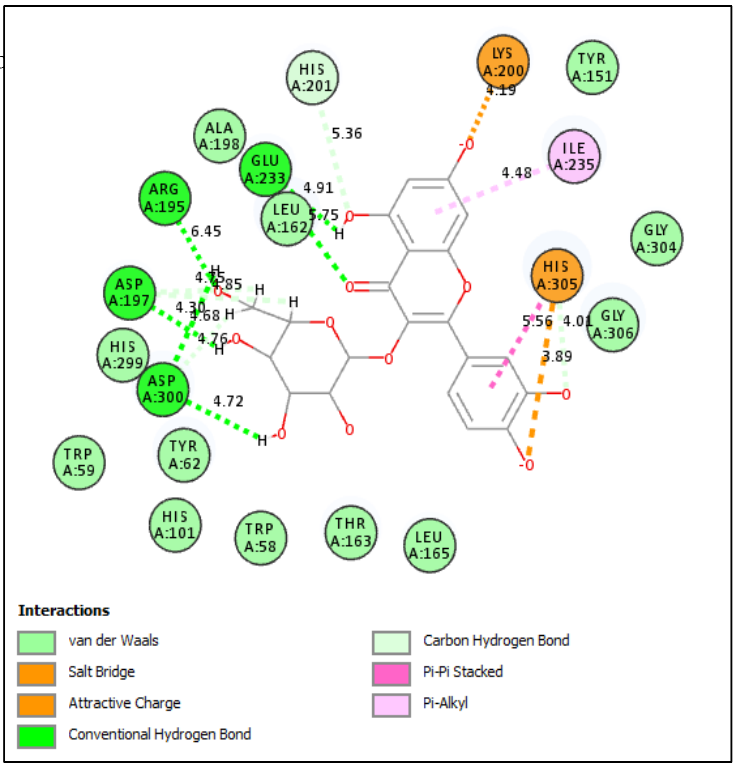

Isoquercitrin (4)

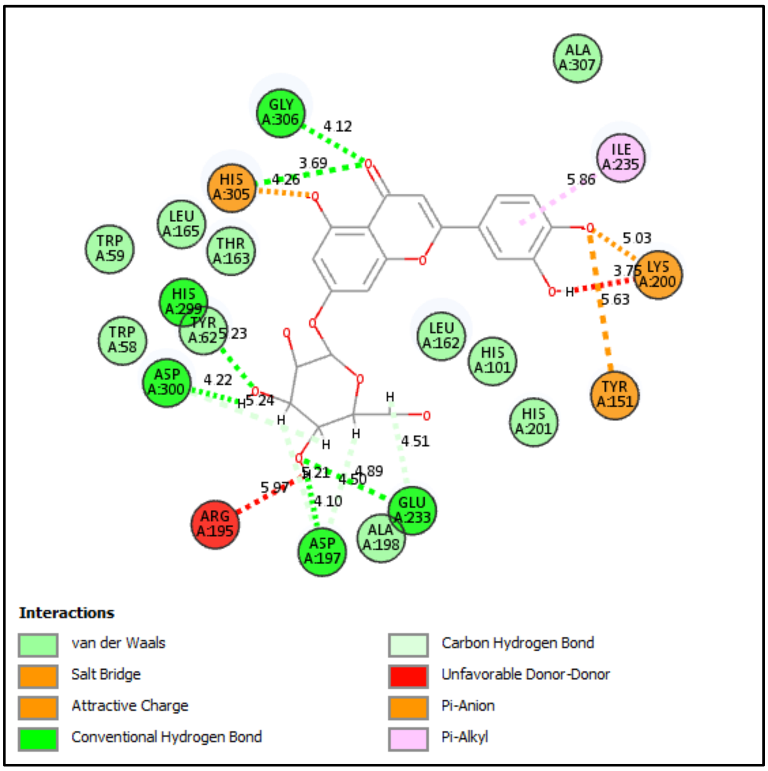

Luteolin 7-O-β-D glucopyranoside (5)

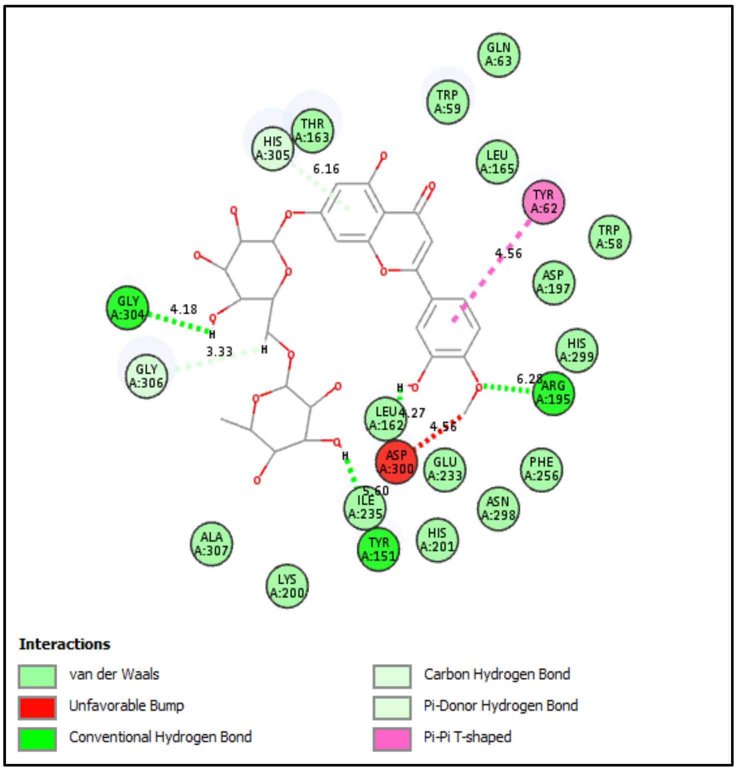

Diosmin (6)

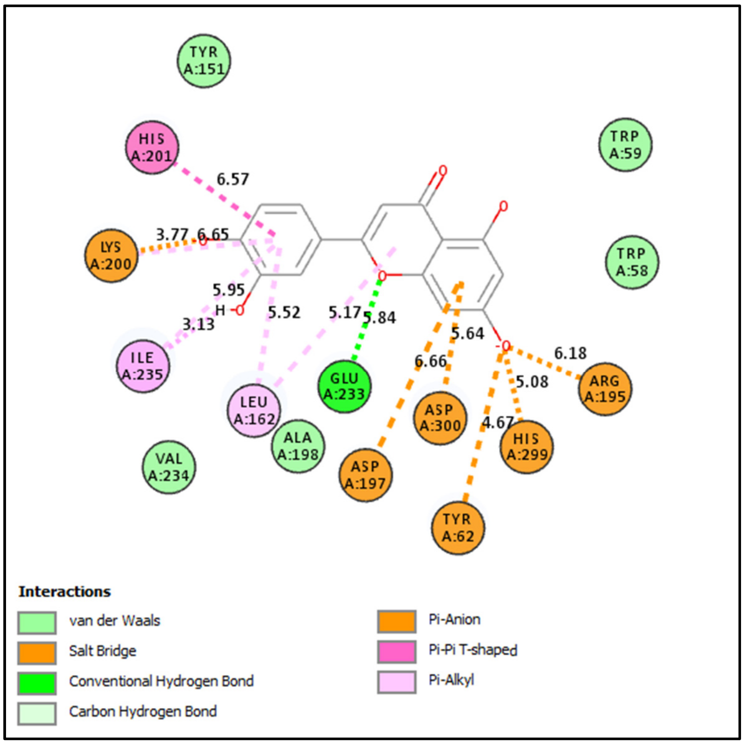

Luteolin (7)

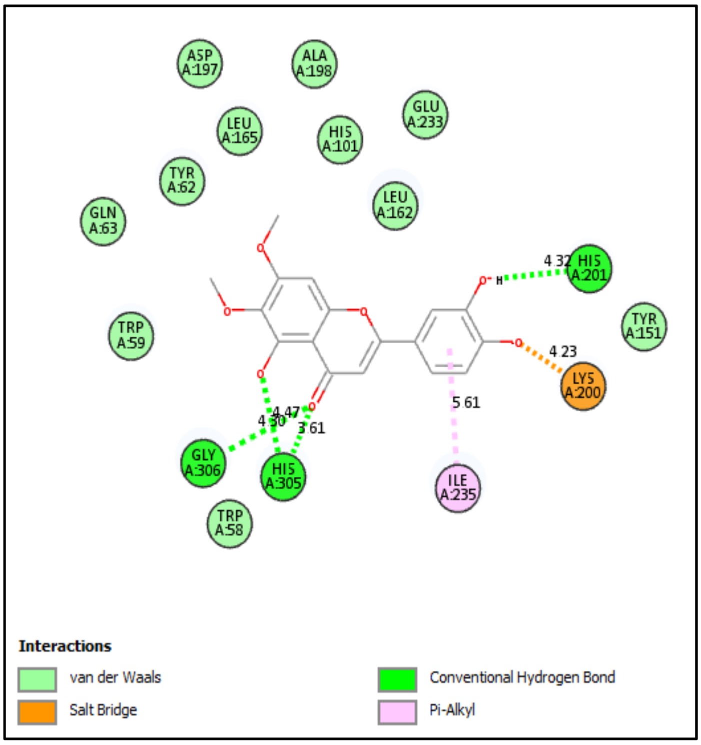

Cirsiliol (8)

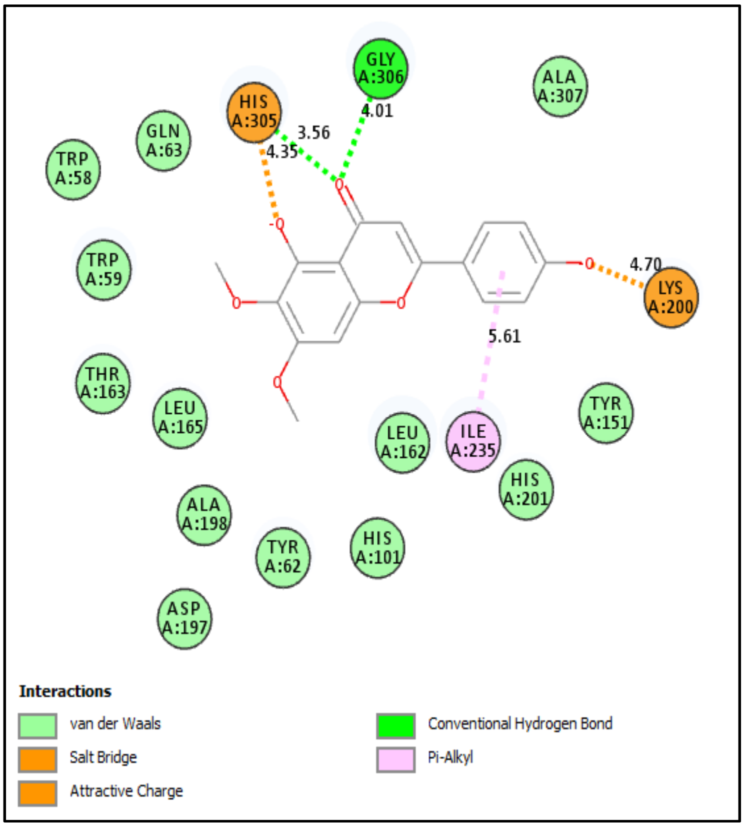

Cirsimaritin (9)

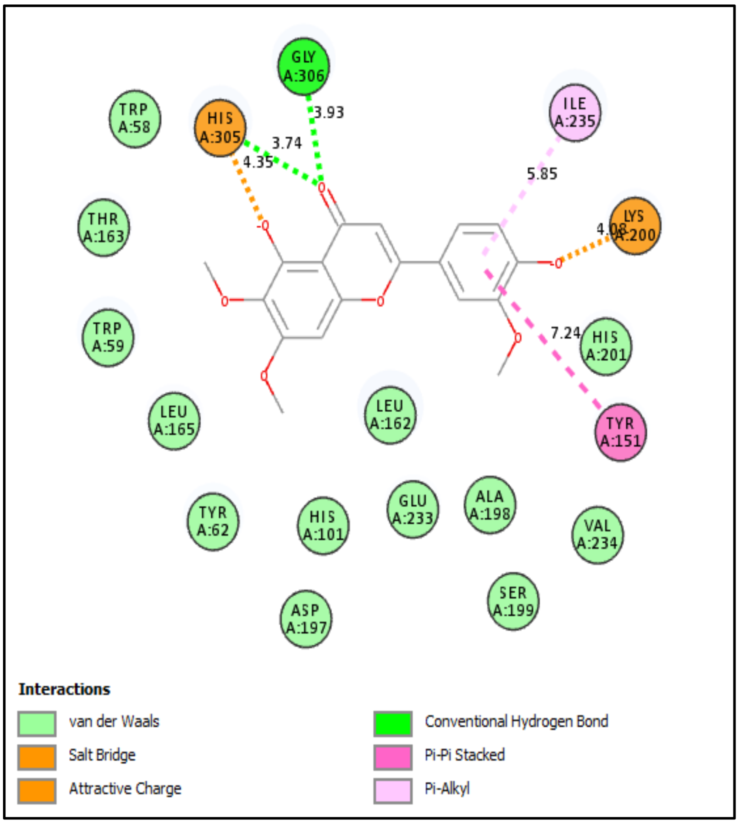

Cirsilineol (10)

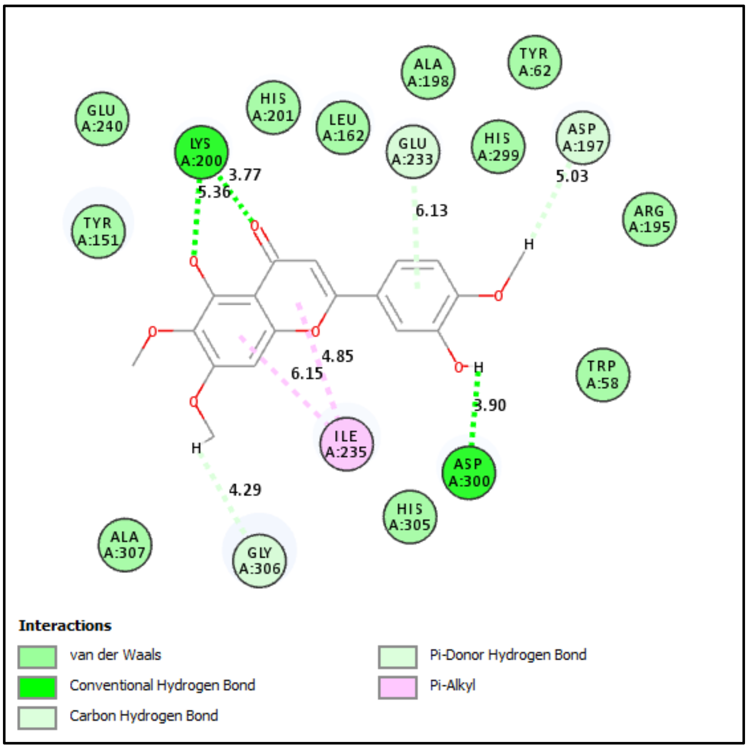

Eupatorin (11)

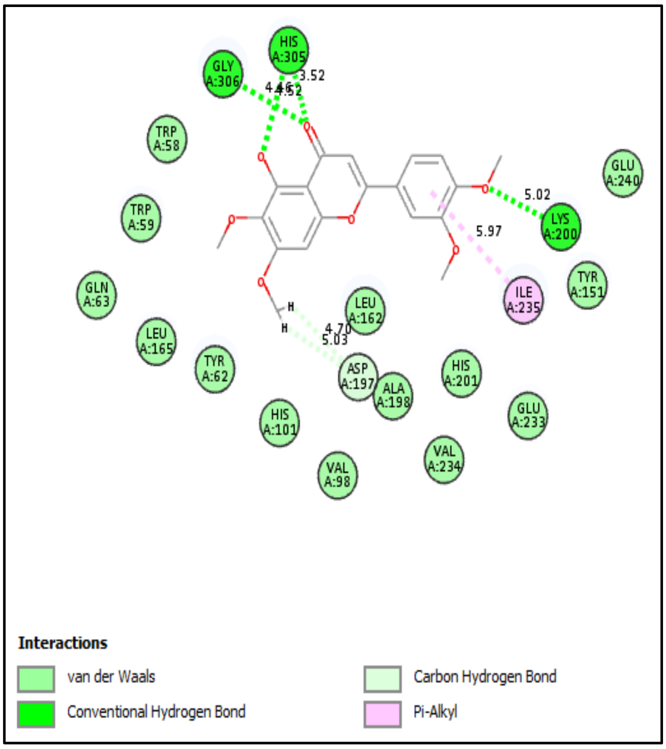

5-Desmethylsinensetin (12)

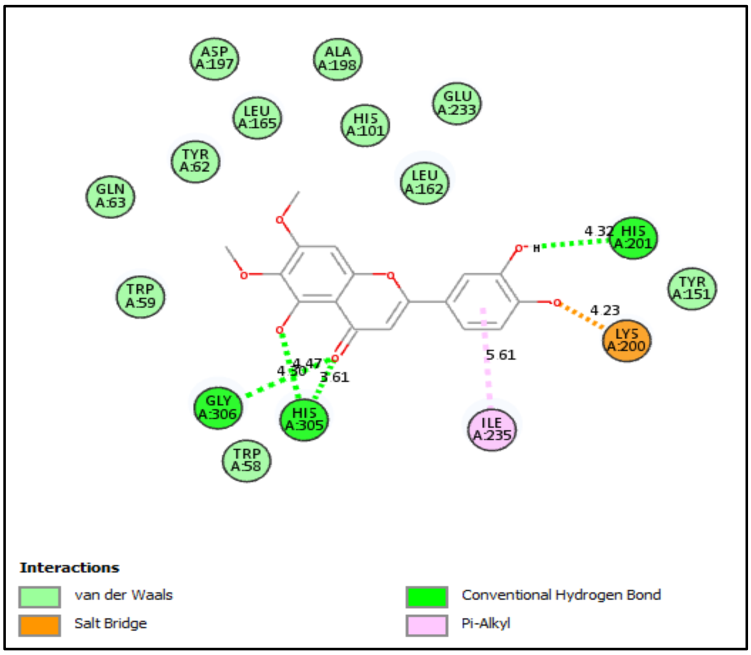

Salvigenin (13)

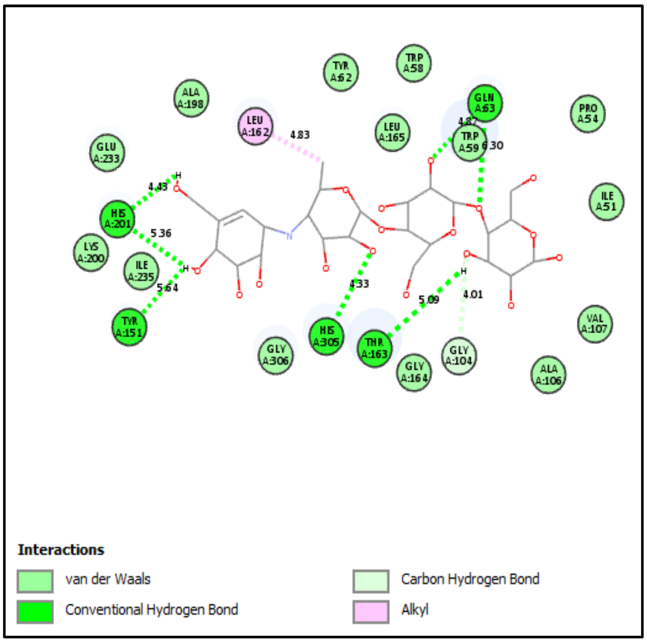

Acarbose

Figure S3: 2D binding mode of compounds identified from Algerian *Teucrium polium* aerial parts inside the active site of 5-lipoxygenase

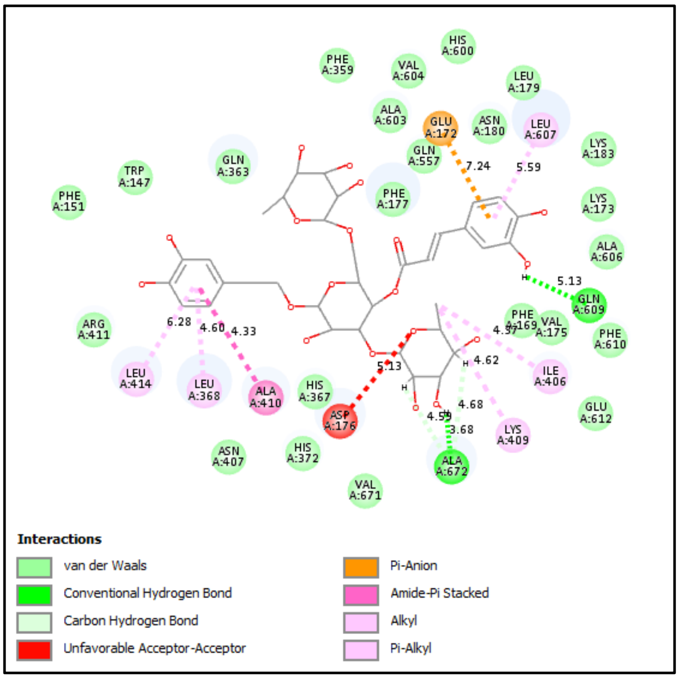

Poliumoside (1)

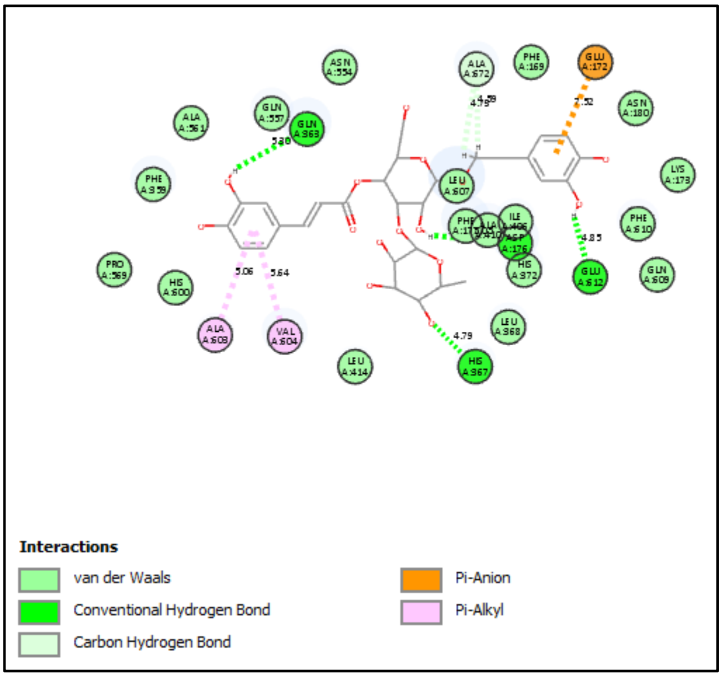

Acteoside (2)

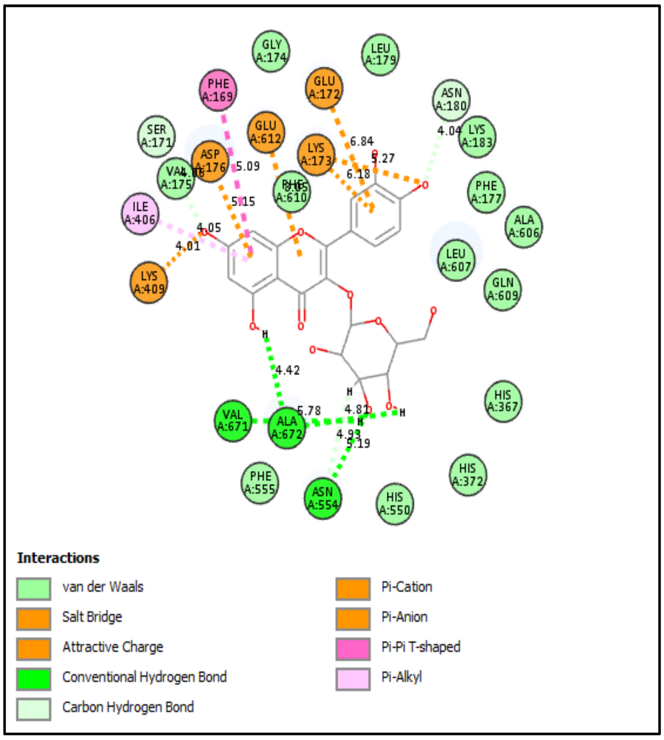

Hyperoside (3)

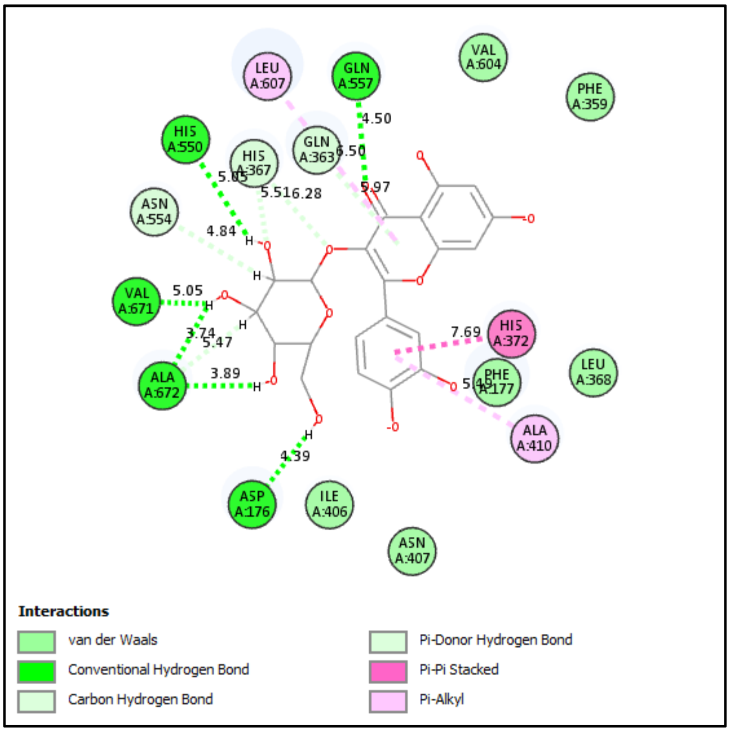

Isoquercitrin (4)

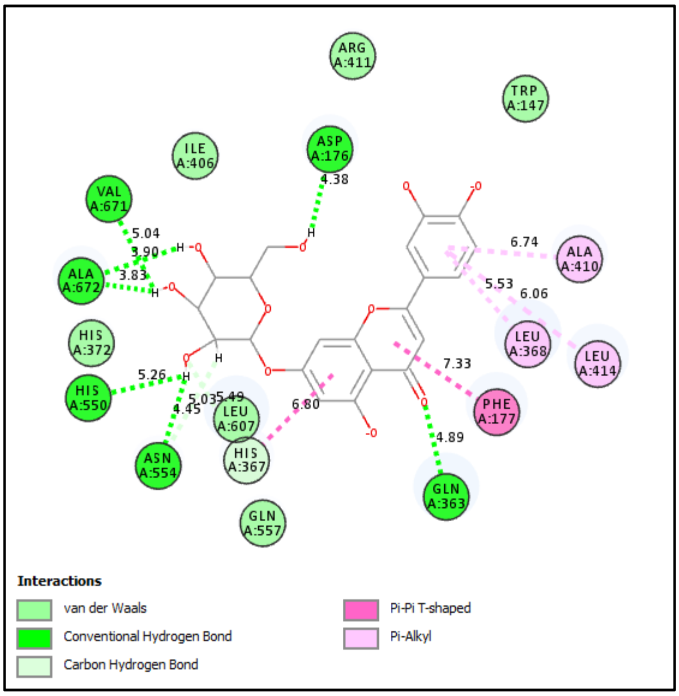

Luteolin 7-O-*β*-D glucopyranoside (5)

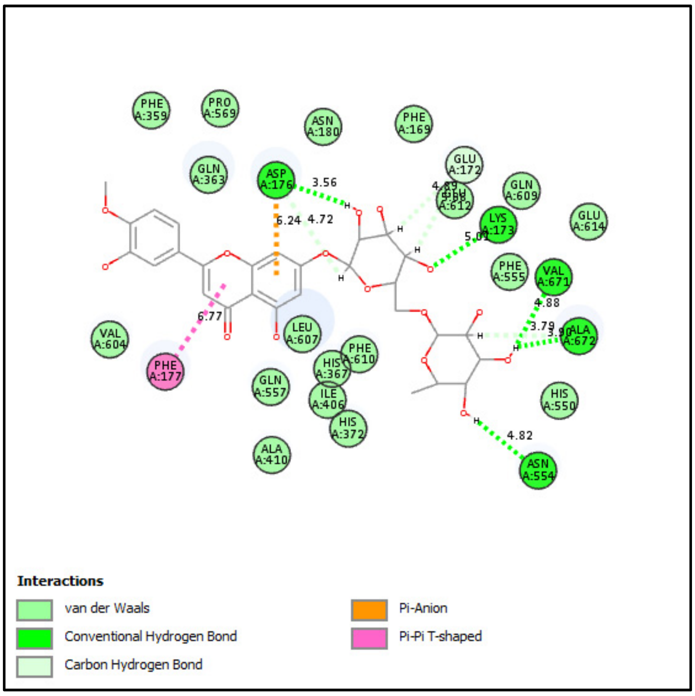

Diosmin (6)

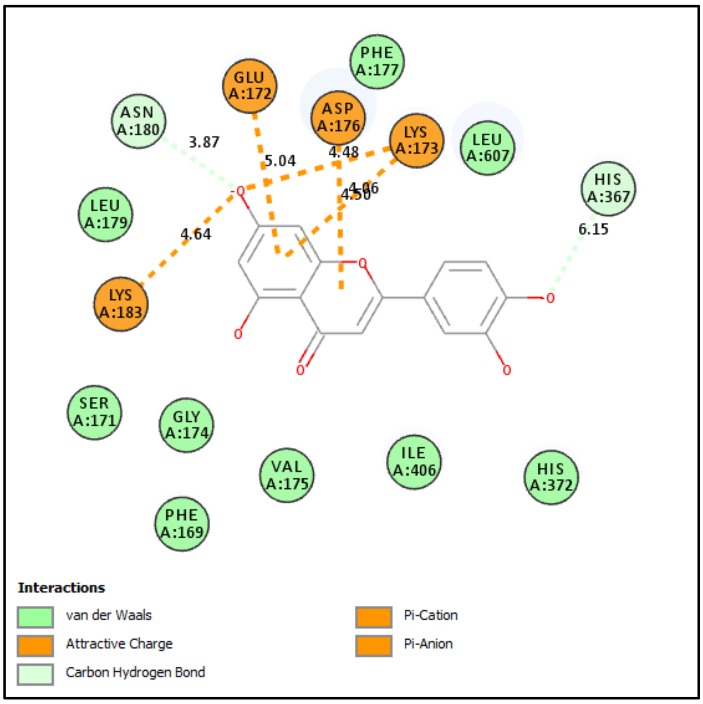

Luteolin (7)

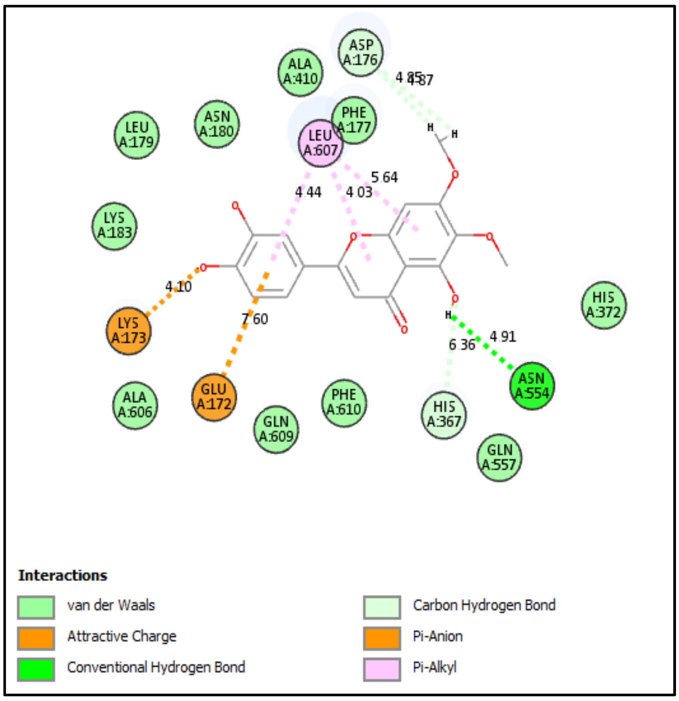

Cirsiliol (8)

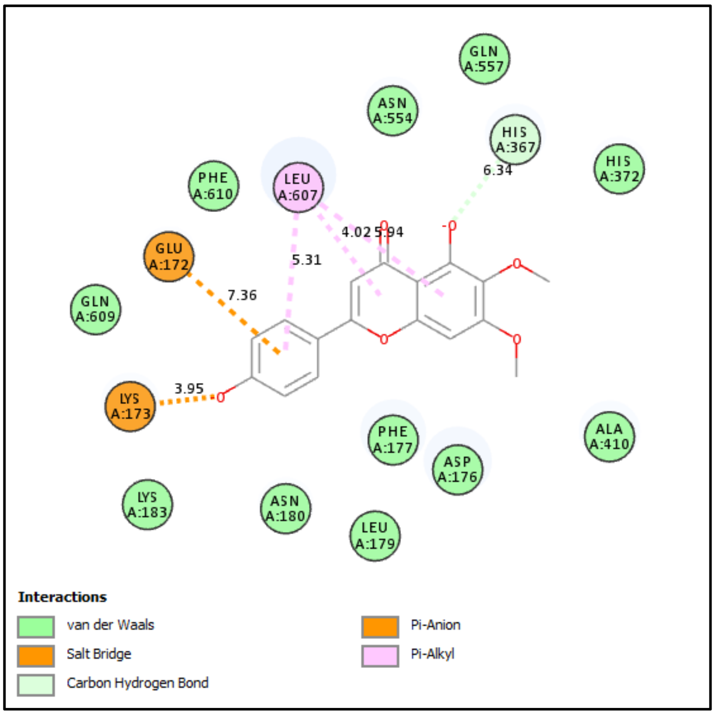

Cirsimaritin (9)

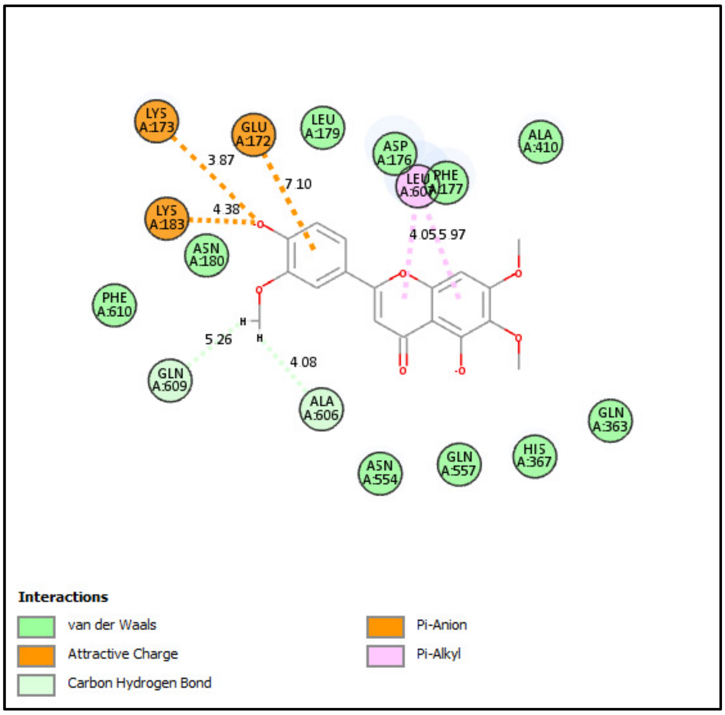

Cirsilineol (10)

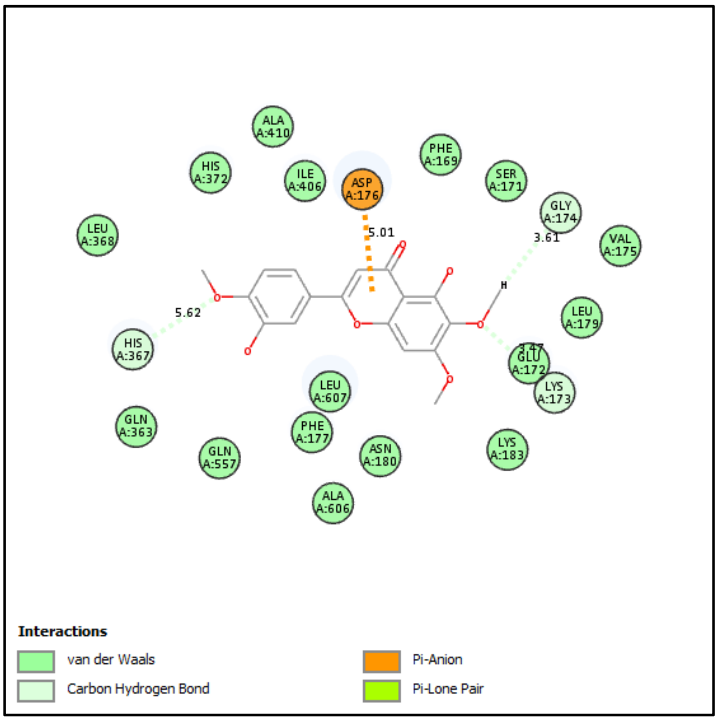

Eupatorin (11)

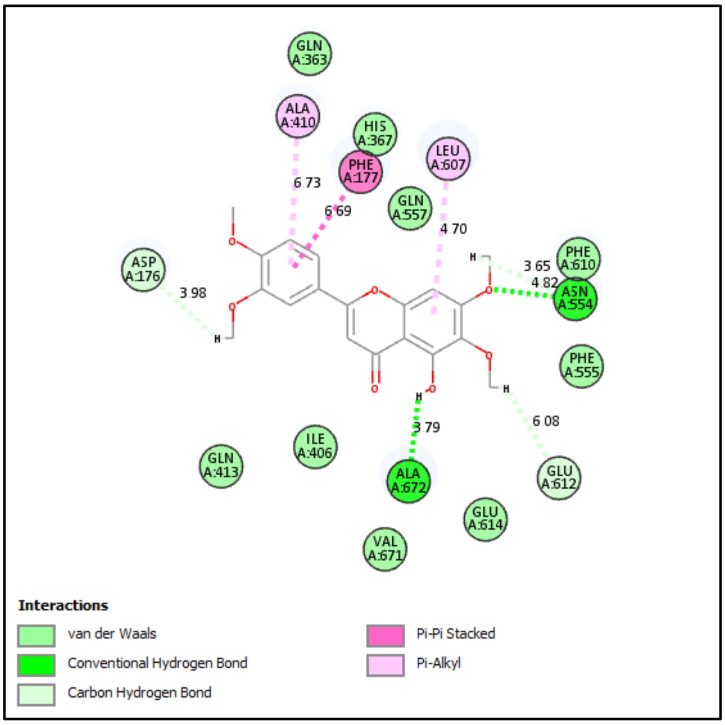

5-Desmethylsinensetin (12)

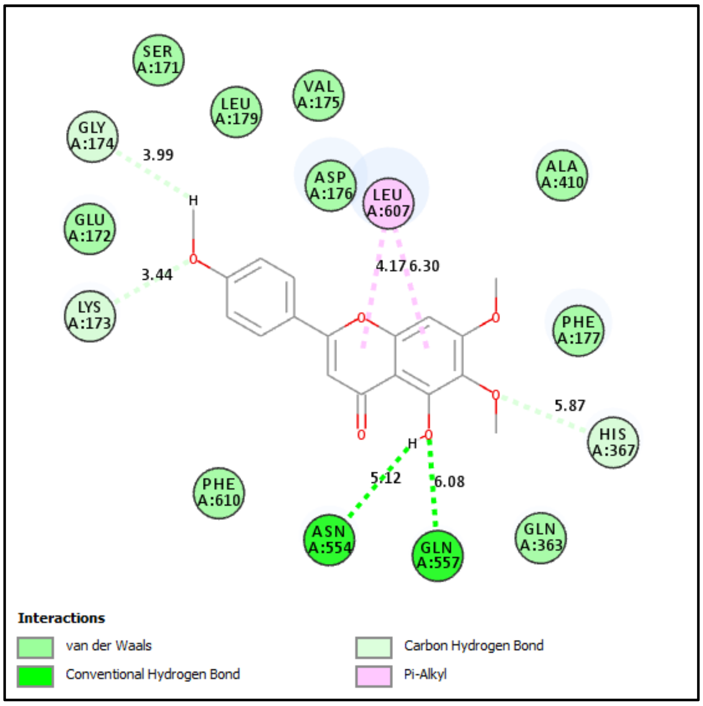

Salvigenin (13)

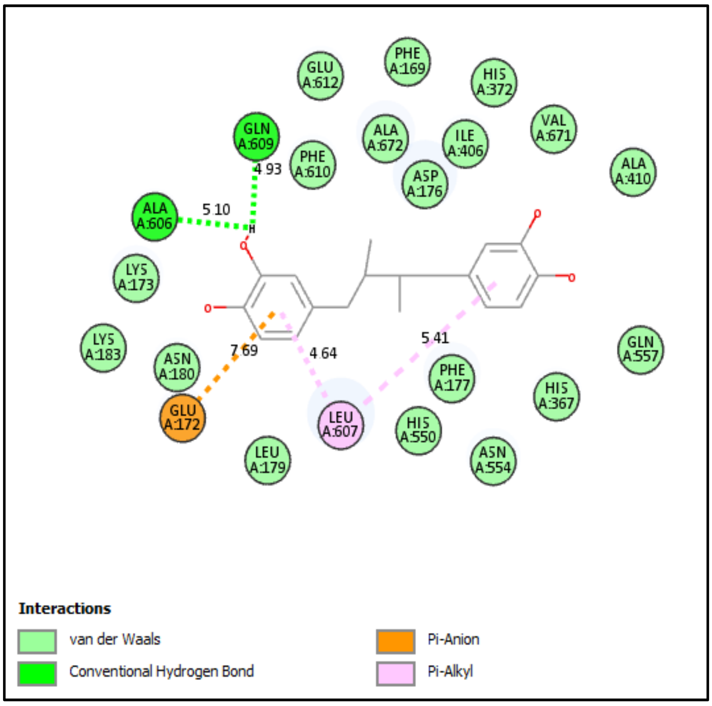

Nordihydroguaiaretic acid

Figure S4: 2D binding mode of compounds identified from Algerian *Teucrium polium* aerial parts inside the active site of acetylcholine esterase

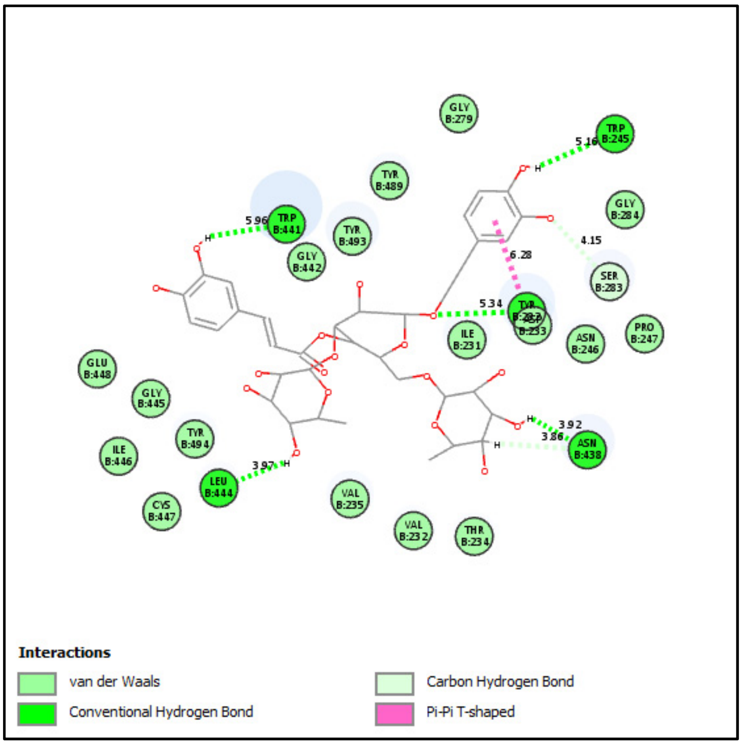

Poliumoside (1)

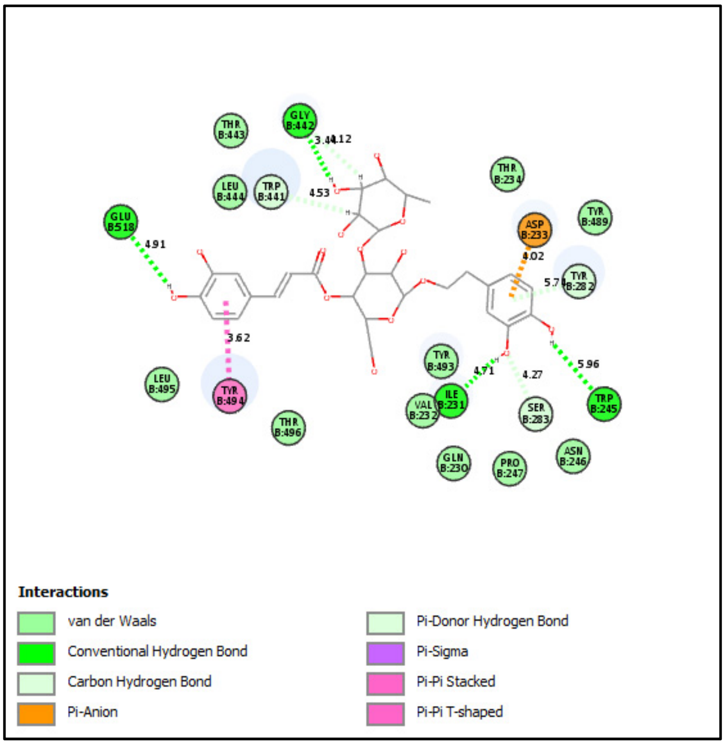

Acteoside (2)

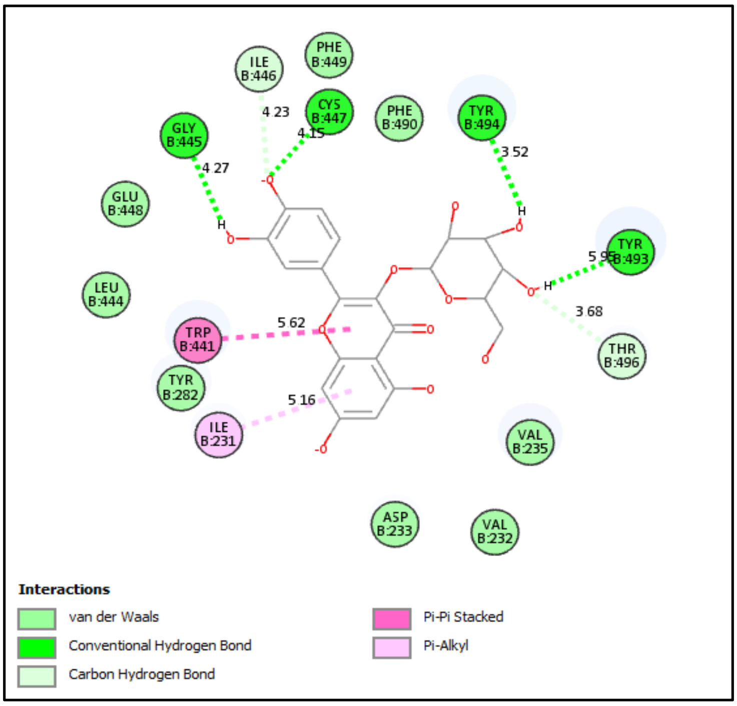

Hyperoside (3)

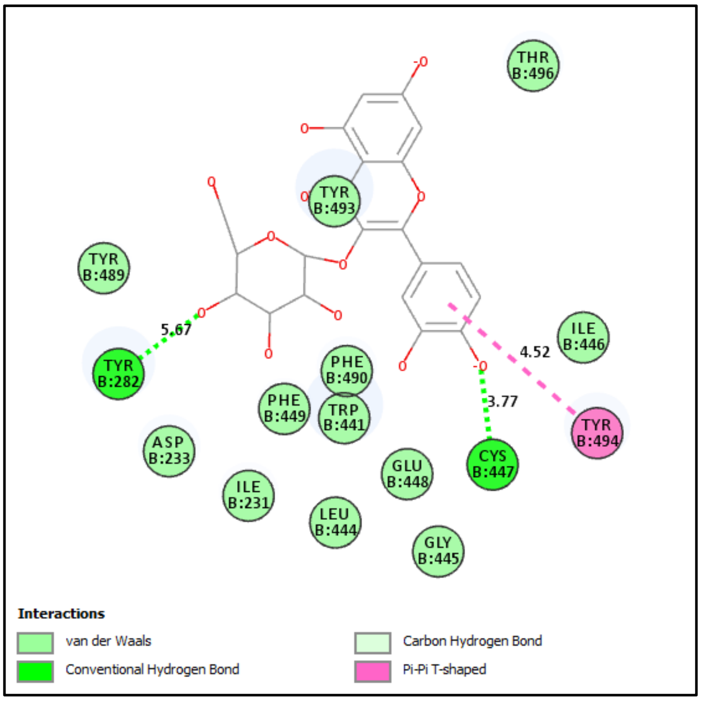

Isoquercitrin (4)

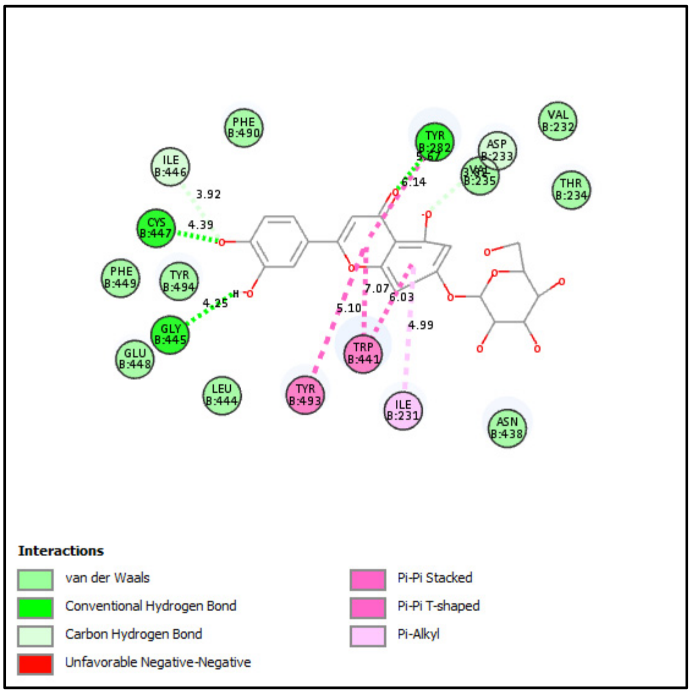

Luteolin 7-O-*β*-D glucopyranoside (5)

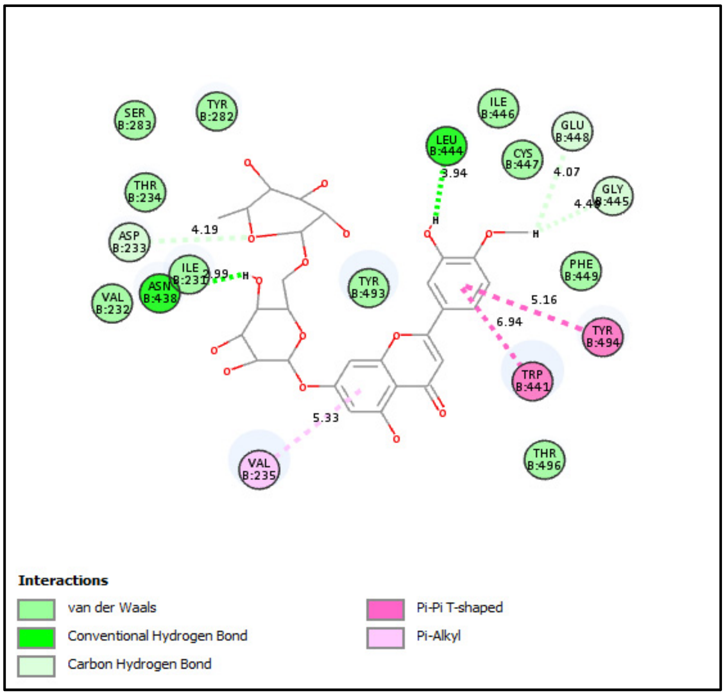

Diosmin (6)

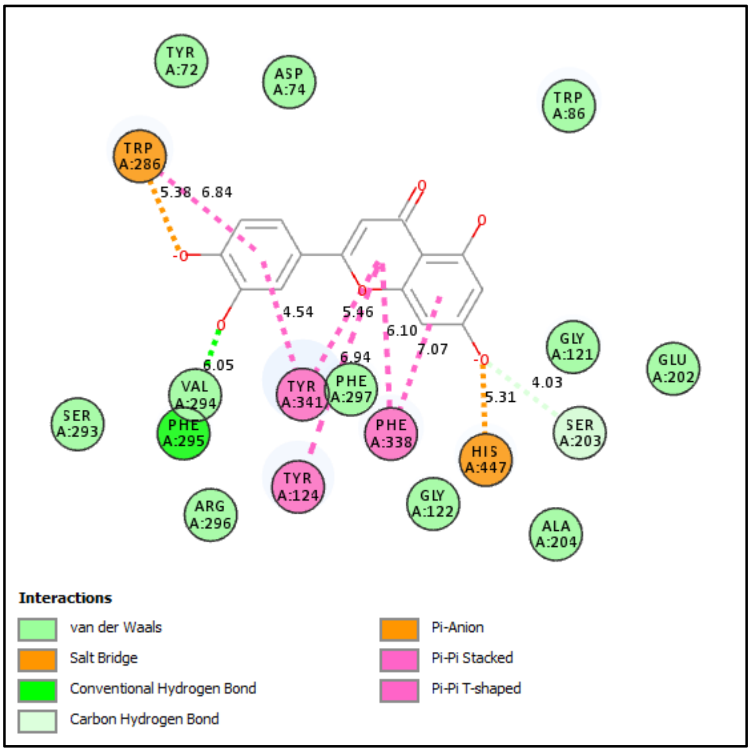

Luteolin (7)

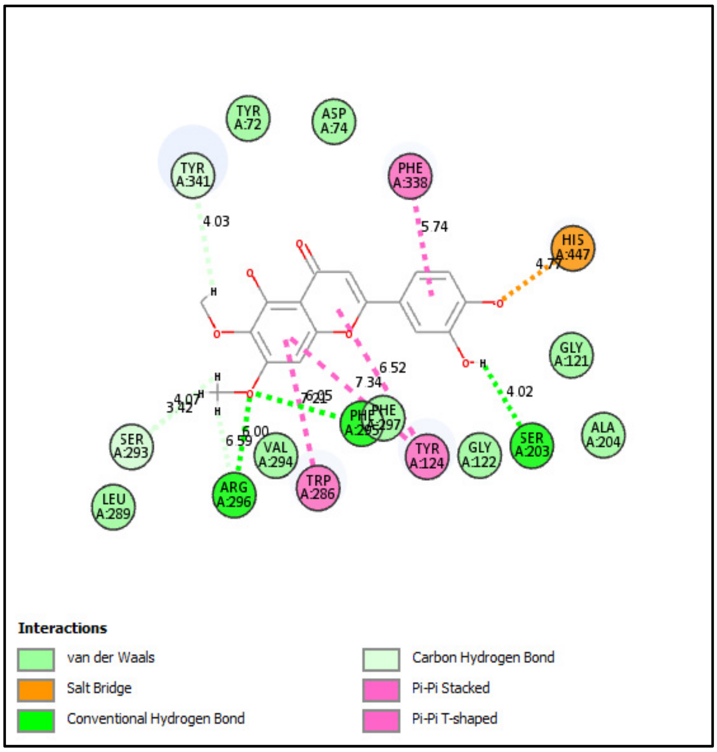

Cirsiliol (8)

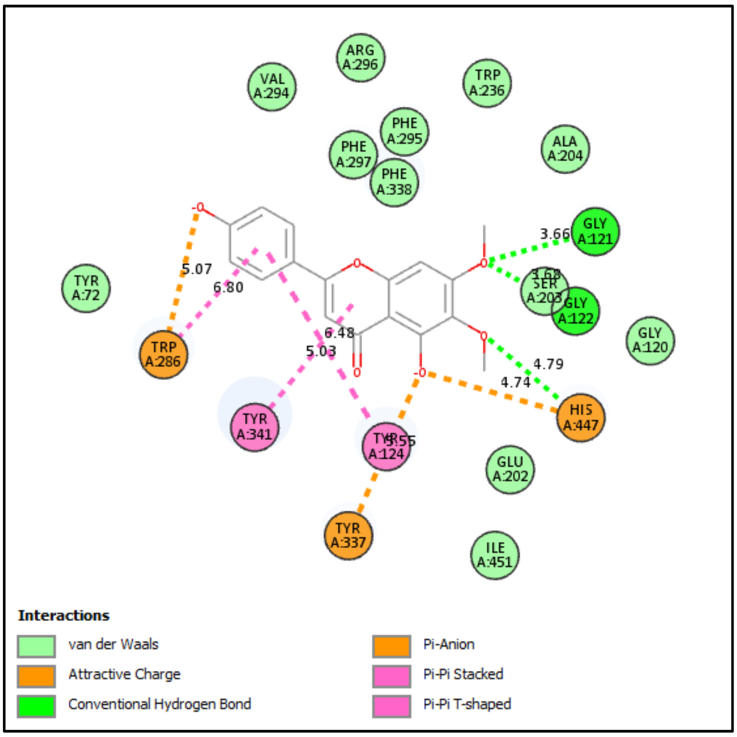

Cirsimaritin (9)

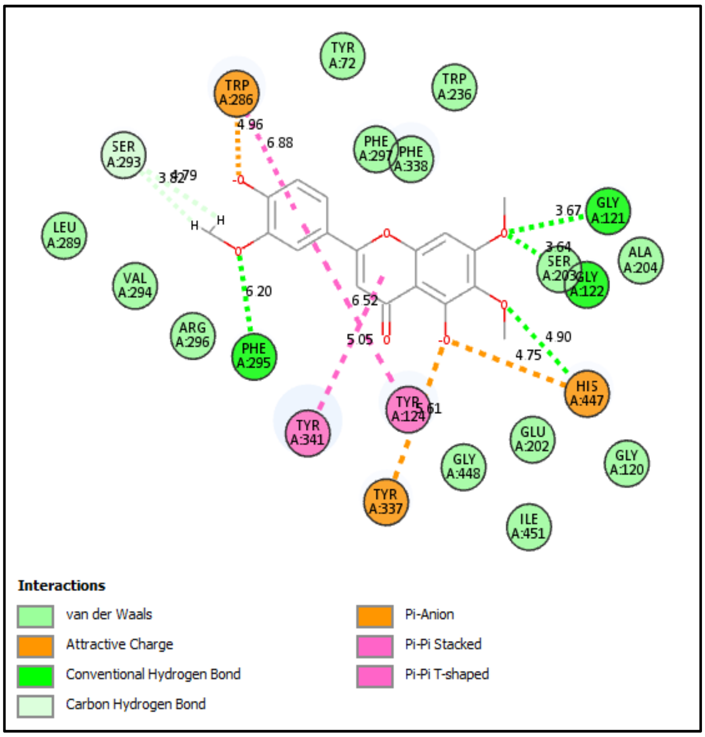

Cirsilineol (10)

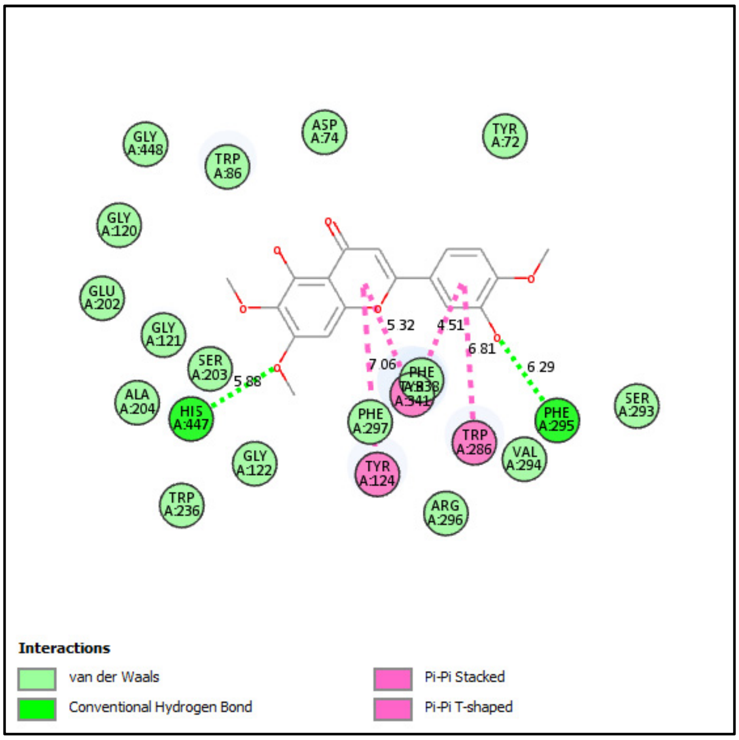

Eupatorin (11)

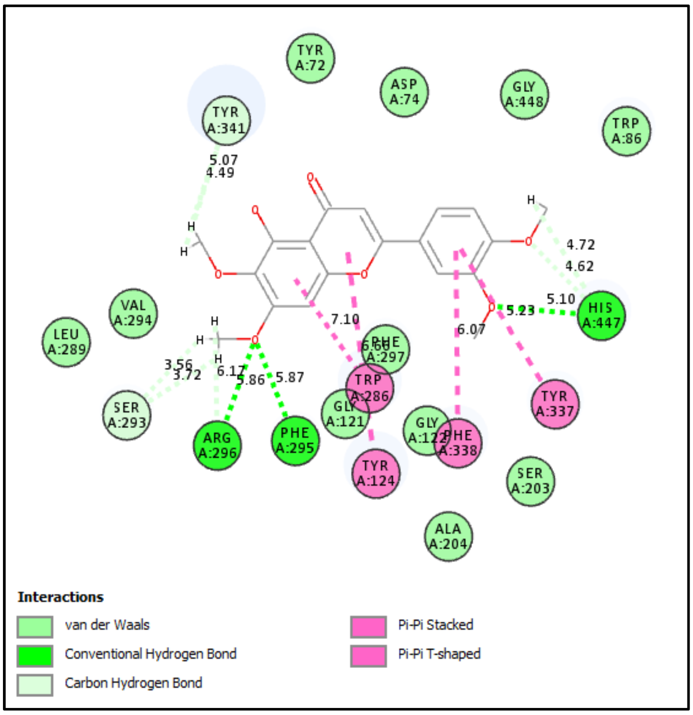

5-Desmethylsinensetin (12)

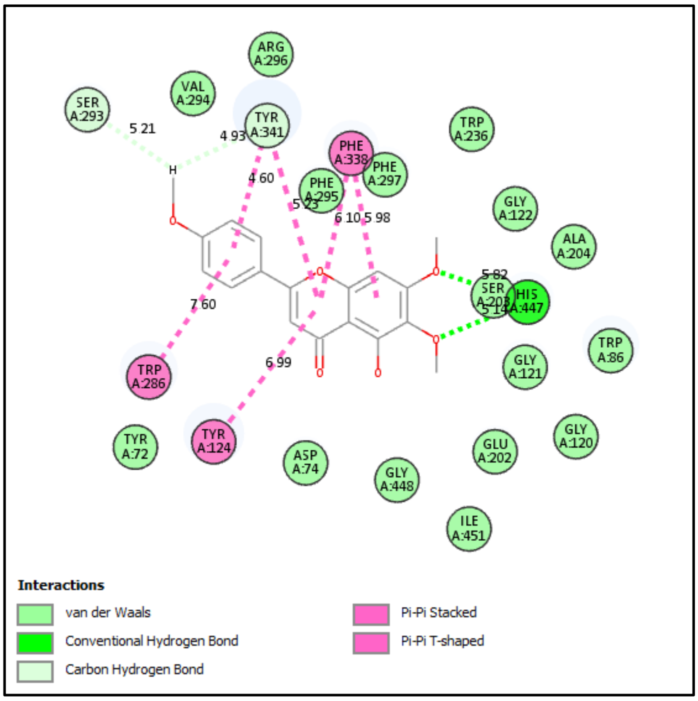

Salvigenin (13)

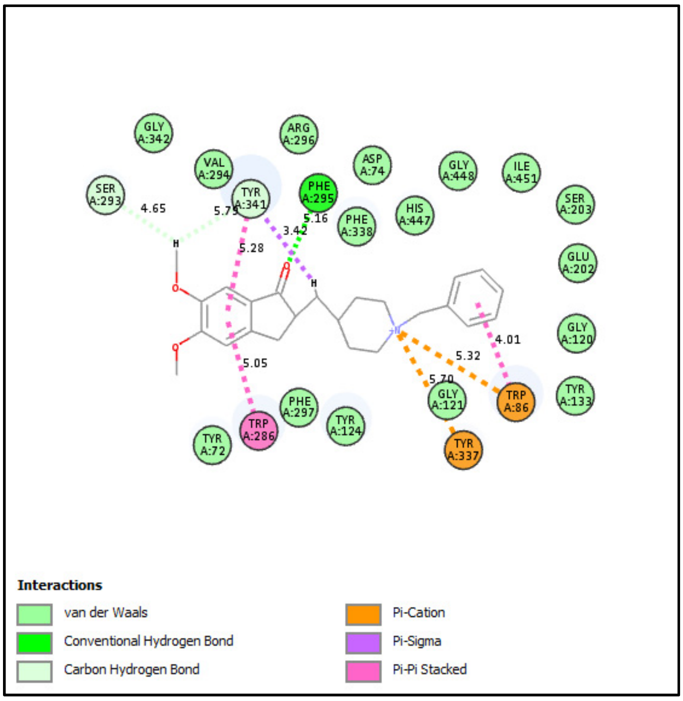

Donepezil
